# Urinary exosomal lnc-TAF12–2:1 promotes bladder cancer progression through the miR-7847–3p/ASB12 regulatory axis

**DOI:** 10.1016/j.gendis.2024.101384

**Published:** 2024-08-05

**Authors:** Song Chen, Jie Cheng, Shuangtai Liu, Danni Shan, Ting Wang, Xinghuan Wang

**Affiliations:** a Department of Urology, Zhongnan Hospital of Wuhan University, Institute of Urology, Wuhan University, Wuhan, Hubei 430071, China; b Department of Cardiovascular Surgery, Zhongnan Hospital of Wuhan University, Wuhan, Hubei 430071, China; c Hubei Provincial Engineering Research Center of Minimally Invasive Cardiovascular Surgery, Wuhan, Hubei 430071, China; d Wuhan Clinical Research Center for Minimally Invasive Treatment of Structural Heart Disease, Wuhan, Hubei 430071, China; e Department of Gastroenterology, Zhongnan Hospital of Wuhan University, Wuhan, Hubei 430071, China; f Department of Biological Repositories, Zhongnan Hospital of Wuhan University, Wuhan, Hubei 430071, China; g Human Genetic Resources Preservation Center of Hubei Province, Wuhan, Hubei 430071, China; h Wuhan Research Center for Infectious Diseases and Cancer, Chinese Academy of Medical Sciences, Wuhan, Hubei 430071, China; i Medical Research Institute, Frontier Science Center of Immunology and Metabolism, Wuhan University, Wuhan, Hubei 430071, China; j TaiKang Center for Life and Medical Sciences, TaiKang Medical School, Wuhan University, Wuhan, Hubei 430071, China

**Keywords:** ASB12, Bladder cancer, Exosome, lnc-TAF12–2:1, miR-7847–3p

## Abstract

Exosomes encompass a great deal of valuable biological information and play a critical role in tumor development. However, the mechanism of exosomal lncRNAs remains poorly elucidated in bladder cancer (BCa). In this study, we identified exosomal lnc-TAF12–2:1 as a novel biomarker in BCa diagnosis and aimed to investigate the underlying biological function. Dual luciferase reporter assay, RNA immunoprecipitation (RIP), RNA pulldown assays, and xenograft mouse model were used to verify the competitive endogenous RNA mechanism of lnc-TAF12–2:1. We found exosomal lnc-TAF12–2:1 up-regulated in urinary exosomes, tumor tissues of patients, and BCa cells. Down-regulation of lnc-TAF12–2:1 impaired BCa cell proliferation and migration, and promoted cell cycle arrest at the G0/G1 phase and cell apoptosis. The opposite effects were also observed when lnc-TAF12–2:1 was overexpressed. lnc-TAF12–2:1 was transferred by intercellular exosomes to modulate malignant biological behavior. Mechanistically, lnc-TAF12–2:1 packaged in the exosomes relieved the miRNA-mediated silence effect on ASB12 via serving as a sponger of miR-7847–3p to accelerate progression in BCa. ASB12 was also first proved as an oncogene to promote cell proliferation and migration and depress cell cycle arrest and cell apoptosis in our data. In conclusion, exosomal lnc-TAF12–2:1, located in the cytoplasm of BCa, might act as a competitive endogenous RNA to competitively bind to miR-7847–3p, and then be involved in miR-7847–3p/ASB12 regulatory axis to promote tumorigenesis, which provided a deeper insight into the molecular mechanism of BCa.

## Introduction

Bladder cancer (BCa) is acknowledged as one of the most common and highest mortality malignancies in the urinary system, with the incidence rate of males ranking 4th among all cancers in the United States.^1^ BCa is under a feature of concealed morbidity. Due to the absence of diagnostic urothelial markers with consistent clinical utility, most patients are diagnosed only when clinical symptoms such as hematuria appear.^2^ BCa is characterized by high rates of recurrence and rapid progression, and once non-muscle-invasive bladder cancer (NMIBC) evolves into muscle-invasive bladder cancer (MIBC), the survival of patients is significantly reduced.^3^ It has been reported that NMIBC accounts for 3/4 of BCa cases with a 5-year survival rate of over 90%, while the remaining 1/4 MIBC cases show a 5-year survival rate of only 60%.^4^^,^^5^ Studies have demonstrated that the urine microenvironment of tumor cells plays an important role in this process,^6^^,^^7^ which is achieved in part by a variety of secretory components (such as exosomes).^8^ Therefore, exploring the role and molecular mechanism of the urine microenvironment in the development of BCa is conducive to identifying more accurate target biomarkers.

Exosomes represent a class of nanoscale extracellular vesicles with diameters of 40–160 nm, which are considered a form of “liquid biopsy”.^9^ It can be secreted by almost all cells and has gradually attracted increasing attention recently. Studies have concluded that exosomes play crucial roles in tumors,^10^ cardiovascular disease,^11^ nervous system disease,^12^ autoimmune disease,^13^ and metabolic diseases^14^ by releasing intracellular “cargos' (such as protein, lipid, RNA, *etc*.). Recently, accumulating evidence has demonstrated that exosomes secreted by tumor cells contribute to tumor development by promoting tumorigenesis, angiogenesis, epithelial–mesenchymal transition, microenvironment remodeling, and immunomodulation.^15^ Although exosomes are confirmed as unique mediator of BCa progression,^16^^,^^17^ the molecular underpinnings of how exosomes are involved in the progression of carcinogenesis have not yet been well elucidated.

Long non-coding RNAs (lncRNAs), consisting of over 200 nucleotides, are a group of nonprotein-coding RNAs that carry out a wide range of biological functions ^18^. lncRNAs participate in the modulation of proliferation, migration, invasion, angiogenesis, and immune escape of various tumors by interacting with DNA, RNA, or protein at the pre-transcriptional, transcriptional, and post-transcriptional levels.^18^^,^^19^ The functions of lncRNAs are strongly associated with their subcellular distribution. The potential for lncRNAs localized in cell nuclei to regulate processes such as chromatin remodeling, transcription regulation, and variable shear regulation is achieved by interacting with RNA-binding proteins at the transcriptional and post-translational levels.^20^ Nevertheless, lncRNAs appear to regulate gene expression and affect the stability of target mRNA or translation efficiency at post-translational levels when fixed in the cytoplasm.^21^ In addition, lncRNAs can act as competitive endogenous RNA (ceRNA) and sponges of miRNAs to obtain the suppression effects of miRNAs and to relieve the inhibition of downstream mRNAs. Zhang et al reported that lncRNA NEAT1 participates in the progression of ferroptosis in hepatocellular carcinoma via the miR-362–3p/MIOX axis.^22^ Zhong et al identified a novel lncRNA OXCT1-AS1 that could regulate the miR-195/CDC25A axis in glioma cell progression.^23^

It is reported that lncRNAs account for 3.36% of the total RNA content from exosomes^24^ and serve as intercellular communication messengers to modulate tumor cell proliferation, invasion, and migration.^25^ With the advancement of sequencing technology, multiple exosomal lncRNAs related to oncogenes and tumor suppressors have been identified in BCa. Exosomal lncRNA LNMAT2 was found to promote the lymphatic metastasis of BCa via recruiting hnRNPA2B1 and enhancing the level of H3K4 trimethylation.^26^ Moreover, it has been demonstrated that normal bladder cells can secrete exosome-derived lncRNA PTENP1 and deliver it to BCa cells, which function as a decoy of miR-17 to regulate PTEN gene expression and inhibit tumor progression.^27^ Now that exosomal lncRNAs are essential regulators of BCa, it is persuasive to identify more novel tumor biomarkers and to broaden the horizon of disease mechanisms, which will be of great advantage for acquiring a deeper understanding of BCa pathogenesis.

Herein, we identified exosomal lnc-TAF12–2:1 in the urine of BCa patients through lncRNA chip sequencing and intended to reveal the potential regulatory mechanism for the first time. Altogether, both *in vivo* and *in vitro* experimental results indicated that exosomal lnc-TAF12–2:1 served as a novel oncogene and might act as a ceRNA by binding to miR-7847–3p and influencing the miR-7847–3p/ASB12 regulatory axis, thus leading to the origination and progression of BCa.

## Materials and methods

### Clinical specimens and cell cultures

This study enrolled 5 cohorts from Zhongnan Hospital of Wuhan University, and the clinicopathological information of these cohorts is listed in Table S1–5. The urine samples of cohort 1 (including 8 BCa patients and 4 healthy volunteers) were used for lncRNA chip sequencing of exosomes, and the urine or tissue samples of other cohorts (cohorts 2, 3, 4, and 5) were used for gene expression detection. The use of all clinical samples such as urine and tissue specimens and clinical information was approved by the Ethics Committee at Zhongnan Hospital of Wuhan University (No. 2021125), and all research procedures were compliant with the Helsinki Declaration.

All cell lines including SV40 immortalized urothelial cells (SV-HUC-1) and human BCa cells (T24, 5637, UMUC3, J82, RT4) were obtained from the Stem Cell Bank, Chinese Academy of Sciences in Shanghai, China. SV-HUC-1, 5637, and T24 cell lines were maintained in RPMI-1640 medium (Gibco, China), UMUC3 cells were cultured in Dulbecco's modified Eagle medium (Gibco, Australia) supplemented with high glucose and 10% fetal bovine serum (Gibco, Australia), J82 cells were cultured in minimum essential medium (Gibco, Australia), and RT4 cells were cultured in McCoy's 5 A medium (Gibco, China). These cell lines were incubated in a humidified incubator (Thermo Scientific, USA) with 5% CO_2_ at 37 °C.

### Exosome extraction and identification

Extraction of exosomes was performed by gradient centrifugation. To isolate and purify exosomes from cells, the medium was replaced by a serum-free medium when T24 cells were cultured to a density of 70%. After continuing the starvation culture for 24–48 h, fifty milliliters of cell supernatant were collected and centrifuged at 3000 *g* for 10 min at 4 °C. The supernatant was moved to a new centrifuge tube and mixed with a 1/4 volume of exosome extract. After storage in a refrigerator at 4 °C overnight, the sample was centrifuged at 10,000 *g* for 30 min. After discarding the supernatant and adding 150 μL of 1 × phosphate buffer saline (PBS) solution for dilution, the exosomes were obtained and preserved at −80 °C for spare use.

Urinary exosomes were isolated and purified according to the isolation protocol. Fifty milliliters of urine were sequentially centrifuged at 300 *g* for 10 min, 2000 *g* for 10 min, and 10,000 *g* for 30 min, and the supernatant was collected after each centrifugation. Then, the supernatant was removed following continuous centrifugation at 100,000 *g* for 90 min at 4 °C, and the remaining precipitate was resuspended in PBS. Finally, urinary exosomes were obtained by centrifugation at 100,000 *g* for 90 min.

We utilized transmission electron microscopy (TEM), nanoparticle tracking analysis, and western blotting to identify exosomes. The morphology of exosomes was observed with TEM, and the diameter of exosomes could be quantified by nanoparticle tracking analysis. Western blotting was used to detect exosome signature proteins such as TSG101, HSP70, and HSP90.

### lncRNA chip sequencing and bioinformatics analyses

lncRNA chip sequencing of total RNA in urine exosomes was entrusted to Obio Technology (Shanghai, China). The up-regulated lncRNAs were defined as those with a fold change >2 and *p*-value <0.05. To select the most differentially expressed lncRNAs, we took the intersection between two genetic datasets: one was a comparison of BCa patients and normal healthy controls, and the other was a comparison of low-stage and high-stage BCa patients. Possible target genes of lnc-TAF12–2:1 were predicted by the TargetScan (http://www.targetscan.org), starBase (https://starbase.sysu.edu.cn/), microRNA (http://www.microrna.org), miRWalk (http://mirwalk.umm.uni-heidelberg.de/), and microT-CDS (https://www.biostars.org/p/143874/) databases.

### Transfection and construction of stable cell lines

siRNAs (*lnc-TAF12-2:1-si-1*, *lnc-TAF12-2:1-si-2*, *lnc-TAF12-2:1-si-3*, *ASB12-si-1*, *ASB12-si-2*, *ASB12-si-3*), control-siRNA (NC), lnc-TAF12–2:1 overexpression plasmid, and miR-7847–3p mimics were synthesized and purchased from GenePharma Gene Co., Ltd., Suzhou, China. *sh-lnc-TAF12-2:1*, *lv-lnc-TAF12-2:1*, and *shNC* (LV3, LV5) were synthesized by GenePharma Gene Co., Ltd., Suzhou, China. The sense sequence of *lnc-TAF12-2:1-si-*1/sh*-lnc-TAF12-2:1* was 5'-CCUAUUGGUCAGGACCUAATTUUAGGUCCUGACCAAUAGGTT-3', the sense sequence of *lnc-TAF12-2:1-si-2* was 5-CCUUUAGAACUGAAGCUAUTTAUAGCUUCAGUUCUAAAGGTT-3', the sense sequence of *lnc-TAF12-2:1-si-3* was 5'-GCUGUAAUCCCAGCACUUUTTAAAGUGCUGGGAUUACAGCTT-3, the sense sequence of *ASB12-si-1* was 5'-CCACUUGAGCUGUUUGCAATTUUGCAAACAGCUCAAGUGGTT-3', the sense sequence of *ASB12-si-2* was 5'-GCAUCAAACAUAGCUUCAUTTAUGAAGCUAUGUUUGAUGCTT-3', the sense sequence of *ASB12-si-3* was 5'-GCCAGCCACAAGCCAUCAATTUUGAUGGCUUGUGGCUGGCTT-3', the sense sequence of control-siRNA/*shNC* (LV3, LV5) was 5'-UUCUCCGAACGUGUCACGUTT-3', and the sense sequence of miR-7847–3p mimics was 5'-CGUGGAGGACGAGGAGGAGGCCUCCUCCUCGUCCUCCACGUU-3'. lnc-TAF12–2:1 cDNA (1561 bp) was polymerase chain reaction (PCR) amplified from a cDNA library of human BCa cell lines and then cloned into a pcDNA3.1 (+) empty vector. Plasmids or siRNA oligonucleotides were transfected into BCa cells using Lipofectamine 2000 (Invitrogen, USA) transfection reagent according to the manufacturer's instructions. In addition, puromycin (5 μg/mL) was used to select and harvest stable cell lines. Infection efficiency was estimated by quantitative real-time PCR (qRT‒PCR) 48 h after infection.

### Exosome cocultivation

A certain number of exosomes from T24 cells was collected as required in the experiment. In a 24-well plate, J82 and UMUC3 cells were cultured overnight and washed three times with fetal bovine serum-free medium. PKH67-Exo labeled with specific fluorescence was added to each well at final concentrations of 5 μg/mL and 10 μg/mL. Subsequently, the cells were incubated at 37 °C for 8 h and 24 h. At the end of the incubation, we discarded the medium and moisturized the cells with PBS three times. The cells were fixed with 4% paraformaldehyde and moisturized with sterile PBS three times after removing the paraformaldehyde, and the cells were treated with 0.1% Triton X-100 PBS for 5 min.

### Cell malignant biological behavior

We investigated the effects of lnc-TAF12–2:1 and related genes on malignant behavior using BCa cells (T24, 5637, UMUC3, J82, RT4), including proliferation assays, migration assays, clonogenic assays, cell apoptosis assays, and cycle assays. A detailed description of the procedure can be found in the supplementary materials and methods.

### qRT‒PCR

A HiPure Total RNA Mini Kit (Cat. #R4111- 03, Magen, China) was used to isolate total RNA from cells and bladder tissues, and a ReverTra Ace qPCR RT Kit (Toyobo, China) was used for reverse transcription. qRT‒PCR was carried out on iQTM SYBR® Green Supermix (Bio-Rad, USA). Fold enrichment was calculated by applying the 2^−ΔΔCt^ method and normalized to GAPDH expression. All sequences of the primer sets used in this study are listed in Table S6.

### lnc-TAF12–2:1 expression level detection in nuclear and cytosolic fractions

Nuclear and cytosolic fractions were extracted by a nuclear-cytosol extraction kit (Ambion, Austin, TX) according to the manufacturer's instructions. A certain number of cells (T24 or 5637 cells) were collected and resuspended in cell fractionation buffer and incubated on ice for 10 min. After centrifugation, the supernatant and nuclear particles were preserved using a cell fragmentation buffer to extract RNA. Relative expression of lnc-TAF12–2:1 was measured in each nuclear and cytoplasmic RNA.

### Western blot

BCa cell lysates and protein samples were prepared with RIPA buffer, protease inhibitor, and phosphatase inhibitor (Sigma–Aldrich, USA). The Bradford protein assay (Bio-Rad, Germany) was used to evaluate the protein concentration. Western blot analysis was performed after the total protein samples were fractionated by 7.5%–15% SDS‒PAGE. Immunoreactive bands were visualized using an enhanced chemiluminescence kit (Bio-Rad, USA) and were then detected using the Molecular Imager ChemiDoc XRS + Imaging System (Bio-Rad, USA). The primary antibodies and secondary antibodies used in this study are listed in Table S7, 8.

### Dual-luciferase reporter assay

According to the complementary sequence of lnc-TAF12–2:1 and miR-7847–3p, the luciferase plasmid of lnc-TAF12–2:1 was constructed, and the wild type (lnc-TAF12-2:1-WT) and mutant lnc-TAF12–2:1 (lnc-TAF12-2:1-MUT) were set up and co-transfected with miR-7847–3p mimics or NC into T24 and 5637 cells. We evaluated the targeting relationship between lnc-TAF12–2:1 and miR-7847–3p by detecting the change in luciferase signal value. A similar strategy was carried out to confirm the relationship between miR-7847–3p and ASB12.

### RNA immunoprecipitation (RIP)

It was proposed to utilize the Magna RIP RNA-binding protein immunoprecipitation kit (Cat. Bes5101, BersinBio, China) for RIP experiments. The details of the procedure are as follows. First, BCa cells were washed and scraped with cold PBS. The cell lysate was prebound with isoantibodies and magnetic beads at 4 °C for 2 h, and the supernatant was used to lyse the cells. Second, 5 μg of primary antibody against the argonaute 2 (AGO2) protein were added and incubated at 4 °C overnight, and anti-biotin magnetic beads were then added and incubated overnight at 4 °C with shaking. Finally, the magnetic beads were washed twice using a cleaning solution with high salt and low salt, and the RNA components were extracted by TRIzol to obtain the purified RNA. lnc-TAF12–2:1 and miR-7847–3p were measured by qRT‒PCR to check whether they were bound to the AGO2 protein.

### RNA pulldown assay

RNA pulldown assays were performed to verify whether miR-7847–3p could bind to lnc-TAF12–2:1 or ASB12. Biotin-labeled lnc-TAF12–2:1 and ASB12 were generated by transcription *in vitro*, and the above RNA was treated with RNase-free DNase I and then purified by a RNeasy Mini Kit (Cat. Bes5204, BersinBio, China). The whole cell lysates of BCa cells were mixed with 3 μg biotin-labeled RNA and incubated at 25 °C for 1 h. Then, Dynabeads™ M−270 streptavidin was made available for enrichment and purification. Finally, TRIzol was used to extract and purify RNA and the expression of miR-7847–3p was detected with qRT‒PCR.

### Xenograft mouse model

Sixteen BALB/c-nu nude mice (male, four weeks old) were divided into four groups, including the lnc-TAF12–2:1 silencing group (sh-lnc-TAF12–2:1), silencing control group (LV3), lnc-TAF12–2:1 overexpression group (LV-lnc-TAF12–2:1), and overexpression control group (LV5). Then, approximately 1 × 10^6^ T24 cells were injected into each mouse. The tumor size and average weight were measured every three days. After continuous measurement for 36 days, the nude mice were anesthetized and sacrificed for further comparison.

T24 cells (3 × 10^6^ cells/mouse) were used to construct a subcutaneous tumor-forming model in nude mice (*n* = 10). When the tumor was visible to the naked eye (50 mm^3^), 10 BALB/c-nu nude mice aged 4 weeks were randomly divided into the experimental group and control group. Exosomes overexpressing lnc-TAF12–2:1 were injected into the tumors of the experimental group mice, while the same volume of PBS was injected into the control group. The tumor size and average weight of nude mice were measured as before, and the morphology of tumor cells was measured by hematoxylin-eosin staining. The expression of lnc-TAF12–2:1, ASB12, and other genes was measured with qRT‒PCR. The changes in ASB12, Ki-67, and N-cadherin in anatomical tumor tissue were measured by immunohistochemistry. The method was placed in the supplementary materials and methods.

### Statistical analysis

Data from three individual experiments are presented as mean ± standard deviation. Continuous data were compared by two-sample *t*-tests, and categorical variables were analyzed by chi-squared tests to assess the differences in characteristics between BCa patients and healthy controls. A receiver operating characteristic (ROC) curve and the area under the curve (AUC) were calculated to judge the diagnostic efficacy of the exosomal lnc-TAF12–2:1 level in BCa. Linear regression was applied to evaluate the correlation of genes. We utilized SPSS 16.0 and GraphPad Prism 7 to perform all statistical analyses. A heatmap and volcano plot were generated by R version 3.5.0, and a *p*-value <0.05 was regarded as statistically significant.

## Results

### lnc-TAF12–2:1 was significantly up-regulated in BCa patients

**Figure 1:**
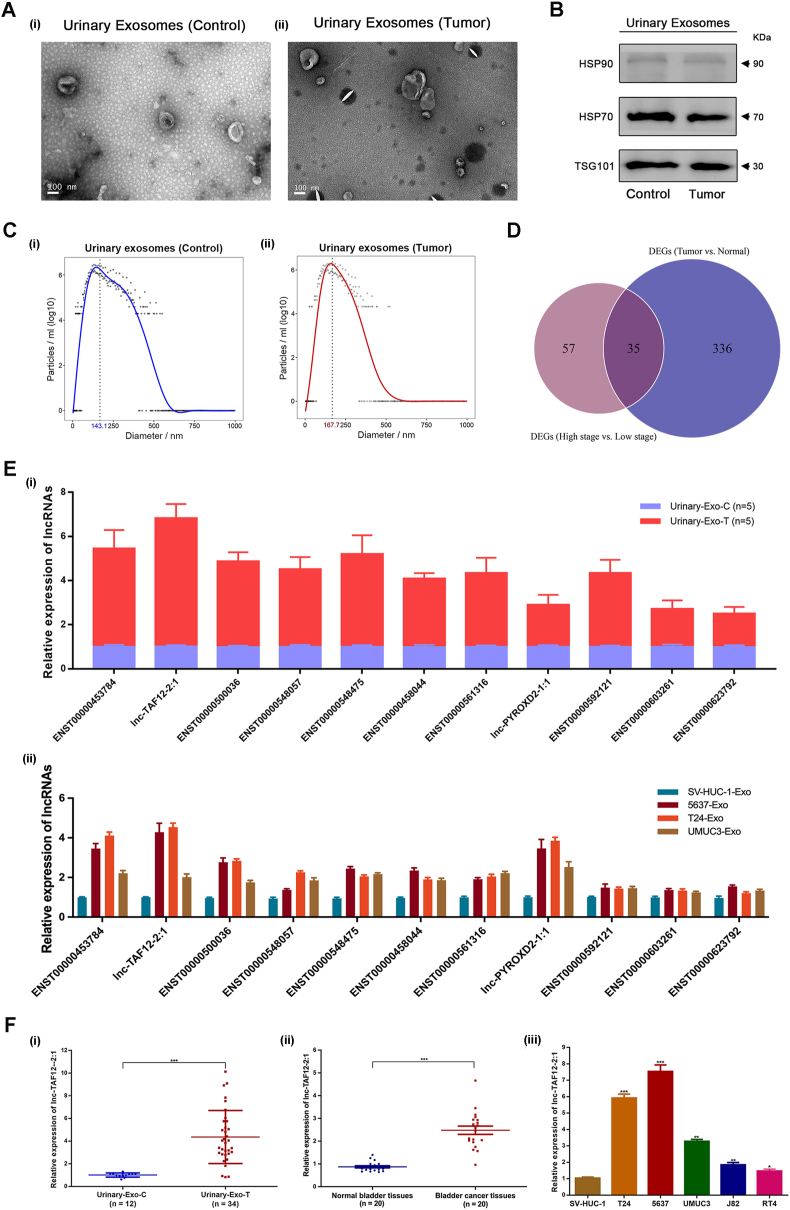
Extraction, identification, high-throughput lncRNA chip sequencing, and differentially expressed lncRNA selection of urinary exosomes isolated from bladder cancer (BCa) patients. **(A)** Transmission electron microscopy (scale bar = 100 nm). **(B)** Western blot. **(C)** Nanoparticle tracking analysis. **(D)** A total of 35 lncRNAs were significantly up-regulated in the intersection datasets of tumor versus normal tissue and high-stage tumor versus low-stage tumor. **(E)** The expression level of the top 11 up-regulated lncRNAs was validated in urinary exosomes from BCa patients (i) and BCa cell lines (ii). **(F)** lnc-TAF12–2:1 was highly expressed in urinary exosomes (i) and tumor tissues of BCa patients (ii), as well as in BCa cell lines (iii).

To search for novel and noninvasive biomarkers for early diagnosis of BCa, we performed lncRNA chip sequencing of urinary exosome RNA collected from 8 BCa patients and 4 healthy volunteers (cohort 1). TEM images (Fig. 1A) showed vesicular morphology, and representative exosome protein markers, such as HSP90, HSP70 and TSG101, were also assessed by western blotting (Fig. 1B). Nanoparticle tracking analysis measured the diameter of urinary exosomes, which was 167.7 nm in the tumor group and 143.1 nm in the control group (Fig. 1C). All the above results confirmed the existence of urinary exosomes.

Differently expressed lncRNAs were acquired according to two analysis methods of chip sequencing results. One was to compare the differently expressed genes between tumor and normal tissues, and another was the comparison between high-stage and low-stage BCa tissues. Ultimately, thirty-five up-regulated lncRNAs related to the initiation and progression of BCa were obtained from the intersection of the two analysis results of differently expressed lncRNAs (Fig. 1D and Table S9). To obtain the definitive target lncRNA, the urinary exosome samples of another 5 patients (cohort 2) and several BCa cell lines were added to validate the top 11 lncRNAs (ENST00000453784, lnc-TAF12–2:1, ENST00000500036, ENST00000548057, ENST00000548475, ENST00000458044, ENST00000561316, lnc-PYROXD2-1:1, ENST00000592121, ENST00000603261, ENST00000623792). The results indicated that lnc-TAF12–2:1 was the most highly expressed lncRNA in exosomes (Fig. 1E). Therefore, we chose lnc-TAF12–2:1 as our target gene for subsequent study. lnc-TAF12–2:1 is a newly discovered lncRNA located on chromosome 1p 35.3, and full-length lnc-TAF12–2:1 in BCa cells was obtained by 5′ and 3′ rapid amplification of cDNA ends (RACE) (Fig. S1A; supplementary materials and methods). Furthermore, lnc-TAF12–2:1 expression was verified to be up-regulated in the urinary exosomes (cohort 3) and tissues (cohort 4) of BCa patients, as well as in BCa cell lines (Fig. 1F).

Similarly, the heatmap (Fig. S1B) and volcano plot (Fig. S1C) displayed the aberrantly expressed lncRNAs in lncRNA chip sequencing and showed that lnc-TAF12–2:1 was up-regulated in the urinary exosomes of BCa patients. The ROC curve (including 68 BCa patients and 20 healthy volunteers; cohort 5) indicated that exosomal lnc-TAF12–2:1 might be a diagnostic biomarker of BCa (AUC = 0.854, 95% confidence interval = 0.750–0.959) (Fig. S2).

### Down-regulation of lnc-TAF12–2:1 expression repressed BCa cell proliferation and migration and promoted cell cycle arrest and cell apoptosis

**Figure 2:**
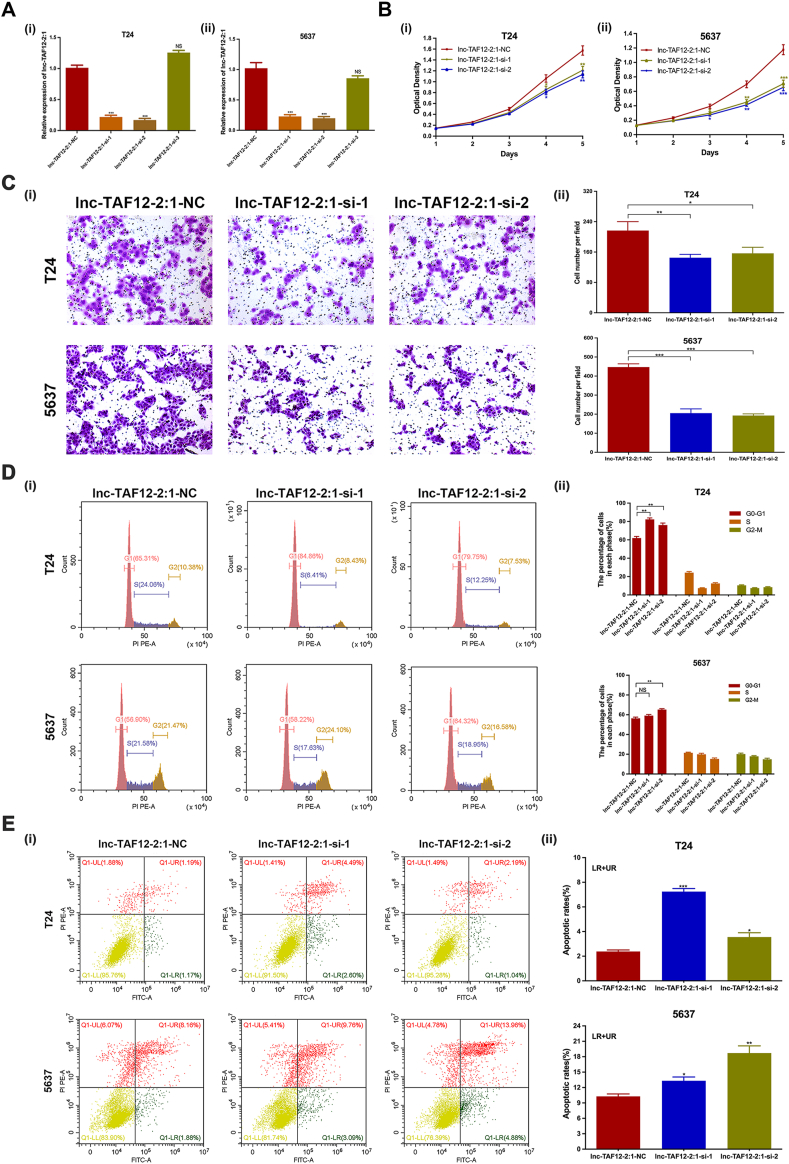
Down-regulation of lnc-TAF12–2:1 expression repressed bladder cancer (BCa) cell proliferation and migration and promoted G0/G1 cell cycle arrest and apoptosis. **(A)** Verification of lnc-TAF12–2:1 siRNA silencing efficacy in T24 (i) and 5637 (ii) cells by quantitative reverse transcription PCR. **(B)** MTT assays indicated that lnc-TAF12–2:1 silencing decreased the proliferation capacity of T24 (i) and 5637 (ii) cells. **(C)** Migration assays depicted that lnc-TAF12–2:1 silencing attenuated cell migration ability. **(D)** lnc-TAF12–2:1 silencing increased cell cycle arrest at the G0/G1 phase. **(E)** lnc-TAF12–2:1 silencing promoted cell apoptosis. ∗*p* < 0.05, ∗∗*p* < 0.01, ∗∗∗*p* < 0.001. NS, no significant.

Knockdown and overexpression functional assays were performed to explore the biological function of lnc-TAF12–2:1 in BCa cells. T24 and 5637 cells were chosen for the following study because of their higher expression of lnc-TAF12–2:1. *lnc-TAF12-2:1-si-1* and *lnc-TAF12-2:1-si-2* showed better knockdown efficacy than *lnc-TAF12-2:1-si-3* at the mRNA level and were used for subsequent experiments (Fig. 2A). MTT assays revealed a significant reduction in cell viability after transfection with *si-lnc-TAF12-2:1* compared with *si-NC* (Fig. 2B), which meant that lnc-TAF12–2:1 silencing could reduce cell proliferation. The migratory capacity of T24 and 5637 cells was dramatically attenuated when lnc-TAF12–2:1 was silenced (Fig. 2C). Moreover, cell cycle experiments demonstrated that knockdown of lnc-TAF12–2:1 increased the proportion of cells arrested in the G0/G1 phase in T24 and 5637 cells (Fig. 2D). Flow cytometry assessment indicated that lnc-TAF12–2:1 silencing promoted cell apoptosis (Fig. 2E). In contrast, up-regulation of lnc-TAF12–2:1 expression could promote proliferation and migration and reduce G0/G1 cycle arrest and cell apoptosis of BCa cells (Fig. S3).

### Intercellular transmission and biological effect of exosomal lnc-TAF12–2:1

**Figure 3:**
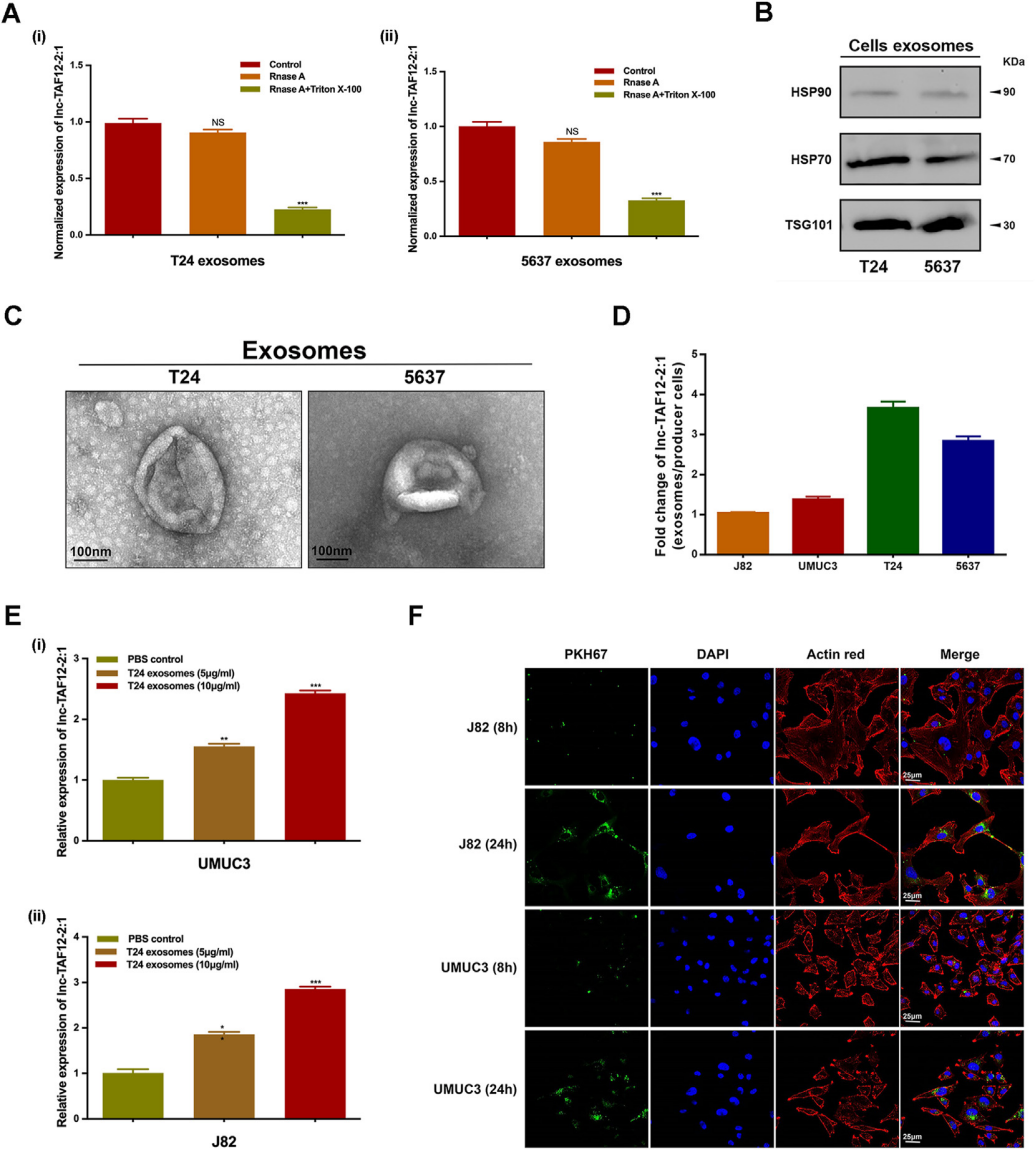
Exosomal lnc-TAF12–2:1 intercellular transmission in bladder cancer (BCa) cells. **(A)** lnc-TAF12–2:1 expression in exosomes treated with RNase A alone or combined with Triton X-100 in T24 (i) and 5637 (ii) cells. Identification of exosomes in T24 and 5637 cells according to Western blot. **(B)** and transmission electron microscopy. **(C)**. Scale bar = 100 nm. **(D)** Relative expression of lnc-TAF12–2:1 in exosomes of BCa cell lines (J82, UMUC3, T24, 5637). **(E)** Expression level of lnc-TAF12–2:1 in UMUC3 and J82 cells after coculturing with different concentrations (5 μg/mL, 10 μg/mL) of exosomes extracted from T24 cells. **(F)** Fluorescence *in situ* hybridization of UMUC3 and J82 cells after coculturing with exosomes isolated from T24 cells for 8 h and 24 h observed by confocal microscopy. Exosomes were labeled with PKH67. Scale bar = 25 μm ∗*p* < 0.05, ∗∗*p* < 0.01, ∗∗∗*p* < 0.001; NS, no significant.

First, we confirmed that exosomal lnc-TAF12–2:1 was successfully isolated from T24 and 5637 cells by performing qRT‒PCR, western blotting, and TEM. The results showed that when treated with RNase A alone, the lnc-TAF12–2:1 level remained constant in exosomes, while when combined with Triton X-100, the expression of lnc-TAF12–2:1 decreased dramatically (Fig. 3A). Specific exosome protein markers detected by western blotting (Fig. 3B) and exosome morphology verified by TEM (Fig. 3C) both proved that lnc-TAF12–2:1 was mainly packaged in urinary exosomes.

The relative expression of lnc-TAF12–2:1 in each BCa cell line is shown in Figure 3D. Expression levels of exosomal lnc-TAF12–2:1 in T24 and 5637 cells were higher than those in J82 and UMUC3 cells. Therefore, we chose J82 and UMUC3 cells with lower expression of exosomal lnc-TAF12–2:1 to coculture with different concentrations (5 μg/mL, 10 μg/mL) of exosomes isolated from T24 cells that expressed higher exosomal lnc-TAF12–2:1. The results showed that the expression of lnc-TAF12–2:1 was dramatically elevated with increasing coculture exosome concentration (Fig. 3E). Moreover, confocal microscopy analysis showed that the number of exosomes in J82 and UMUC3 cells increased significantly after both 8 h and 24 h of coculturing with T24 exosomes, and 24 h of coculturing was significantly higher than 8 h (Fig. 3F). These results suggested that exosomal lnc-TAF12–2:1 had the potential for intercellular transfer, which was found to occur in a concentration-dependent and time-dependent manner.

**Figure 4:**
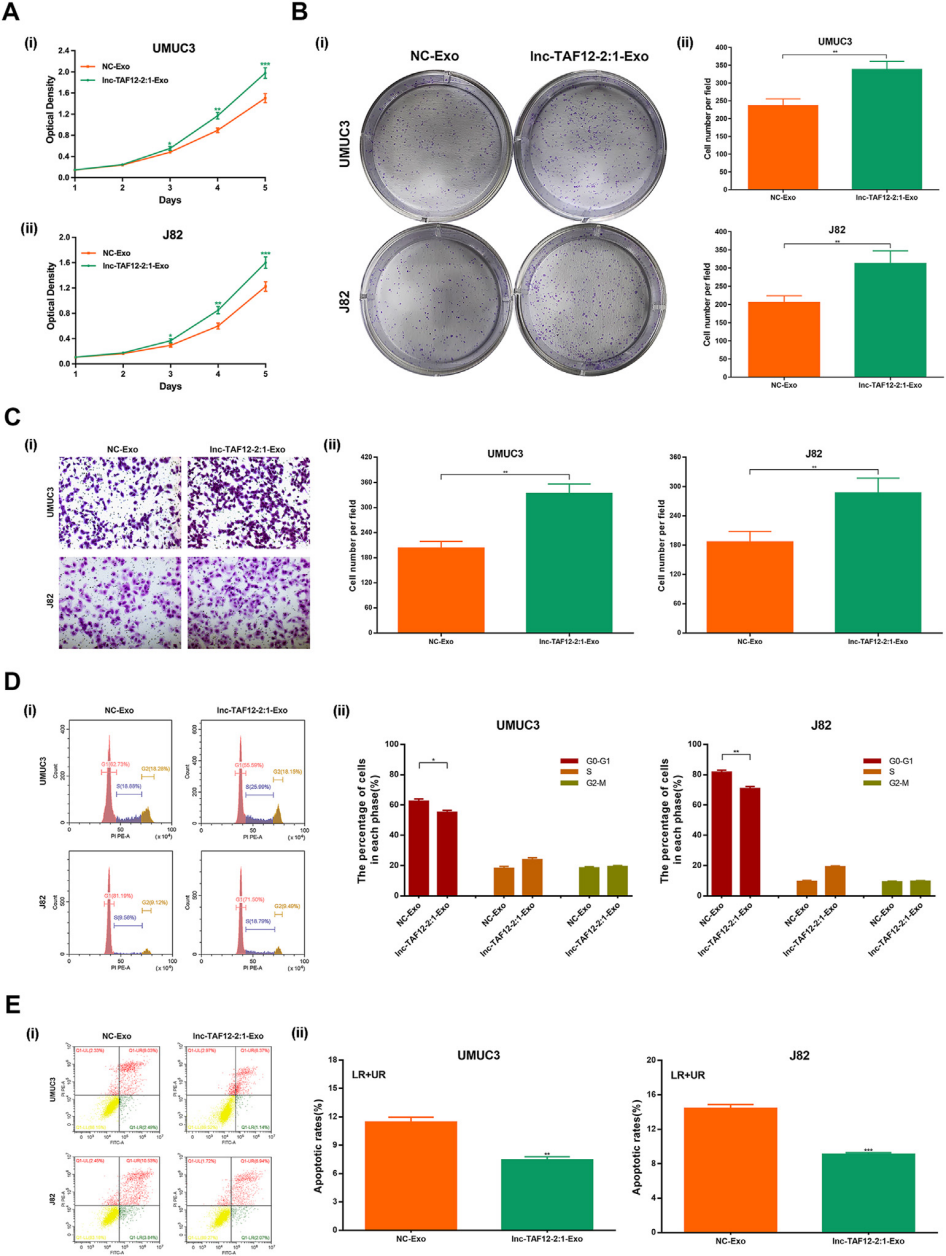
Exosomal lnc-TAF12–2:1 enhanced cell proliferation and migration and reduced cell cycle arrest at the G0/G1 phase and cell apoptosis after transmission. **(A)** MTT assays indicated that coculturing with exosomal lnc-TAF12–2:1 might increase the proliferation capacity of UMUC3 (i) and J82 (ii) cells. **(B)** Clonogenic assays showed that coculturing with exosomal lnc-TAF12–2:1 might increase the proliferation capacity of UMUC3 and J82 cells. **(C)** Migration assays depicted that coculturing with exosomal lnc-TAF12–2:1 might enhance cell migration ability in UMUC3 and J82 cells. **(D)** Coculturing with exosomal lnc-TAF12–2:1 might reduce cell cycle arrest at the G0/G1 phase. **(E)** Coculturing with exosomal lnc-TAF12–2:1 might decrease cell apoptosis. ∗*p* < 0.05, ∗∗*p* < 0.01, ∗∗∗*p* < 0.001.

Afterward, the biological function of exosomal lnc-TAF12–2:1 after coculturing was further clarified. MTT (Fig. 4A) and clonogenic assays (Fig. 4B) revealed that exosomal lnc-TAF12–2:1 could promote cell proliferation in J82 and UMUC3 cells after coculturing with exosomes isolated from T24 cells compared with that in the NC-Exo group. The migration ability of J82 and UMUC3 cells was also increased, assessed by migration assay (Fig. 4C). In addition, exosomal lnc-TAF12–2:1 markedly decreased G0/G1 phase arrest (Fig. 4D) and cell apoptosis (Fig. 4E) compared with the NC-Exo group. These results revealed that exosomal lnc-TAF12–2:1 could play essential biological roles through intercellular transmission, which was essential for mechanism elucidation.

### lnc-TAF12–2:1/miR-7847–3p/ASB12 regulatory axis construction

It is known that the subcellular localization of lncRNAs can determine different biological regulatory roles. To obtain more information about lnc-TAF12–2:1, nuclear and cytosolic fraction assays were employed to verify subcellular localization, and we found that it was mainly situated in the cytoplasm of BCa cells (Fig. S4A). Thus, we attempted to capture potential target miRNAs for lnc-TAF12–2:1 by applying three independent databases (TargetScan, starBase, and microRNA), and capture potential target mRNAs for lnc-TAF12–2:1 by applying another three independent databases (miRWalk, TargetScan, and microT-CDS). As shown in Figure S4B and Table S10, 6 miRNAs (miR-876–3p, miR-3149, miR-4534, miR-1827, miR-7847–3p, miR-548b-3p) were considered to be potential targets of lnc-TAF12–2:1. To search for the most likely target miRNA, qRT‒PCR was conducted to measure the expression changes of miRNAs after transfection with lnc-TAF12–2:1 overexpression or *lnc-TAF12-2:1-si-1* plasmid. We found that miR-7847–3p was the gene with the most obvious changes after up-regulation or down-regulation of lnc-TAF12–2:1 (Fig. S4C). Tumor tissues of cohort 4 were used to explore the correlation between the relative expression of lnc-TAF12–2:1 and miR-7847–3p. A strong negative correlation (*r* = 0.7407, *p* = 0.0002) indicated that miR-7847–3p might be the target miRNA of lnc-TAF12–2:1 (Fig. S4D). The same method was applied to determine the target mRNA of miR-7847–3p. There were 340 common target genes predicted by three independent datasets (Fig. S4E). The expression of ASB12, LY6G5B, MDGA2, VCPKMT, RTN4IP1, ZNF81, CDK12, and ARID2 was consistent with the change in lnc-TAF12–2:1 (Fig. S4F), in which ASB12, VCPKMT, and LY6G5B were also opposite to the expression changes in miR-7847–3p in T24 and 5637 cells (Fig. S4H). Finally, ASB12 was determined because of the highest negative correlation with miR-7847–3p (*r* = −0.6859, *p* = 0.0008) (Fig. S4I).

### lnc-TAF12–2:1 modulated ASB12 expression by interacting with miR-7847–3p

**Figure 5:**
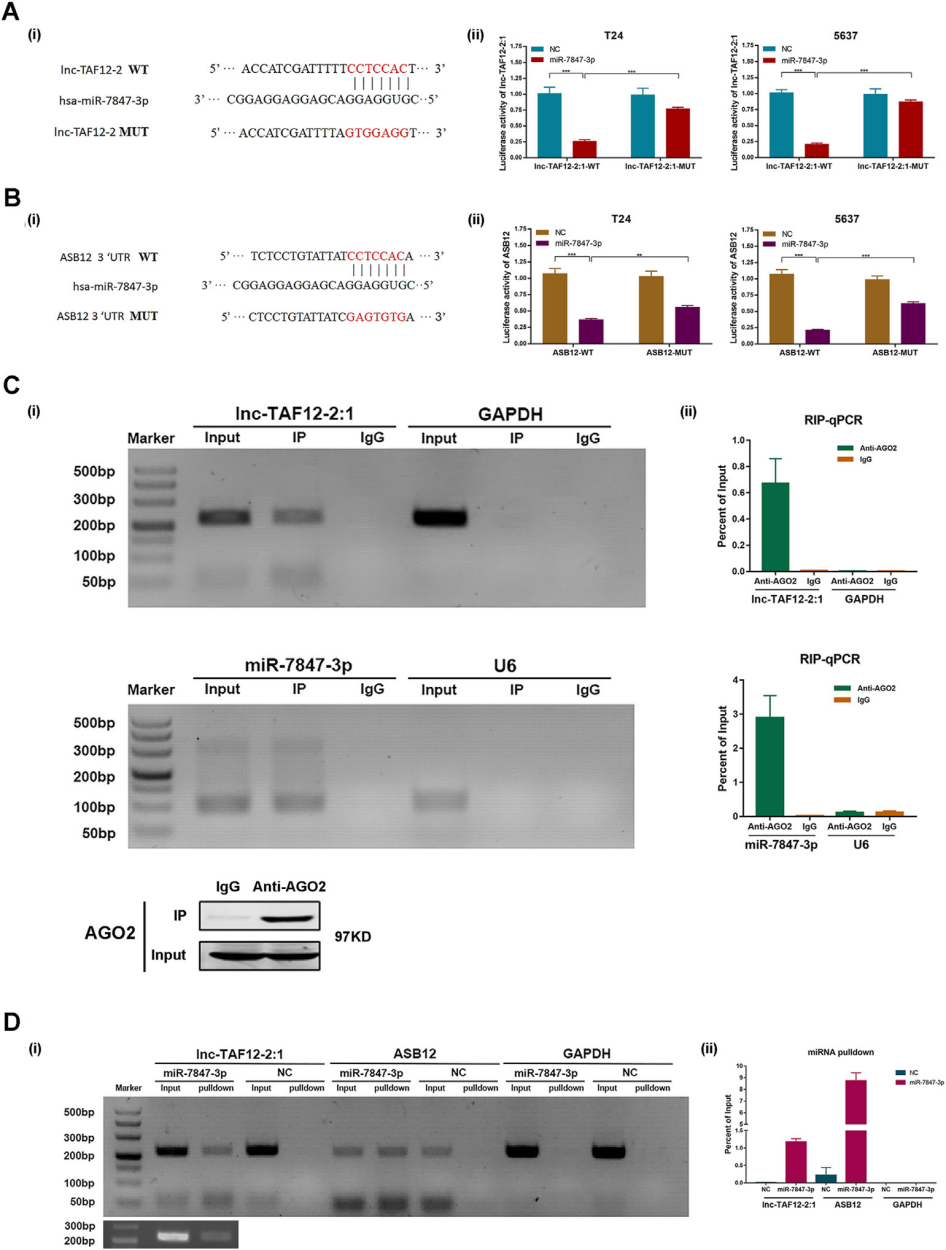
miR-7847–3p bound to lnc-TAF12–2:1 to regulate ASB12 expression. **(A)** Prediction of the binding sites of miR-7847–3p and lnc-TAF12–2:1 from the NCBI database (i). A dual luciferase reporter gene assay verified the target relationship between miR-7847–3p and lnc-TAF12–2:1 (ii). **(B)** Prediction of the binding sites of miR-7847–3p and ASB12 from the NCBI database (i). A dual luciferase reporter gene assay verified the target relationship between miR-7847–3p and ASB12 (ii). **(C)** RNA immunoprecipitation assay was performed using an anti-argonaute 2 antibody to coprecipitate with lnc-TAF12–2:1 and miR-7847–3p. **(D)** RNA pulldown assays were conducted to investigate the interaction of miR-7847–3p with lnc-TAF12–2:1 and ASB12.

We next explored the roles of lnc-TAF12–2:1 in the regulation of the miR-7847–3p/ASB12 axis. Figure 5Ai and 5Bi present the binding site of miR-7847–3p with lnc-TAF-12–2:1 and ASB12, respectively. Then, dual luciferase reporters containing wild type (WT) and mutated type (MUT) of lnc-TAF12–2:1 and ASB12 with binding sites of miR-7847–3p were constructed. The results showed that luciferase activity was reduced when lnc-TAF12-2:1-WT was co-transfected with miR-7847–3p mimics. However, the same phenomenon was not observed in lnc-TAF12-2:1-MUT (Fig. 5Aii), which implied that lnc-TAF12–2:1 might have the ability to interact with miR-7847–3p. Likewise, co-transfection of ASB12-WT and miR-7847–3p mimics showed significantly lower luciferase activity than ASB12-MUT, which also indicated the combination relationship between ASB12 and miR-7847–3p (Fig. 5Bii).

As lnc-TAF12–2:1 was located in the cytoplasm and had a potential target miRNA, we hypothesized that lnc-TAF12–2:1 might act as a sponge of miR-7847–3p to regulate the expression of ASB12. To verify this hypothesis, a RIP assay using an antibody against AGO2 to coprecipitate with lnc-TAF12–2:1 and miR-7847–3p was performed. We observed that both lnc-TAF12–2:1 and miR-7847–3p could be enriched by the antibody AGO2 (Fig. 5C), which is an essential element of RNA-induced silencing complexes. These results suggested that lnc-TAF12–2:1 and miR-7847–3p might coexist in the RNA-induced silencing complexes of BCa cells.

To test the direct binding ability of miR-7847–3p on lnc-TAF12–2:1 and ASB12, we performed a biotin-coupled miRNA pulldown assay and observed more than eight-fold enrichment of ASB12 and one-fold enrichment of lnc-TAF12–2:1 in the capture of miR-7847–3p compared with NC (Fig. 5D).

In rescue experiments, we also found that down-regulation of miR-7847–3p could rescue ASB12 expression at the level of transcription and translation when co-transfected with down-expressed lnc-TAF12–2:1 (Fig. S5A). Migration assays also showed the most invasive reduction in the lnc-TAF12-2:1-si + miR-7847–3p overexpression group (Fig. S5B). All the results concluded that lnc-TAF12–2:1 could regulate the expression of ASB12 by interacting with miR-7847–3p.

### Down-regulation of ASB12 expression repressed BCa cell proliferation and migration and promoted cell cycle arrest at the G0/G1 phase and cell apoptosis

**Figure 6:**
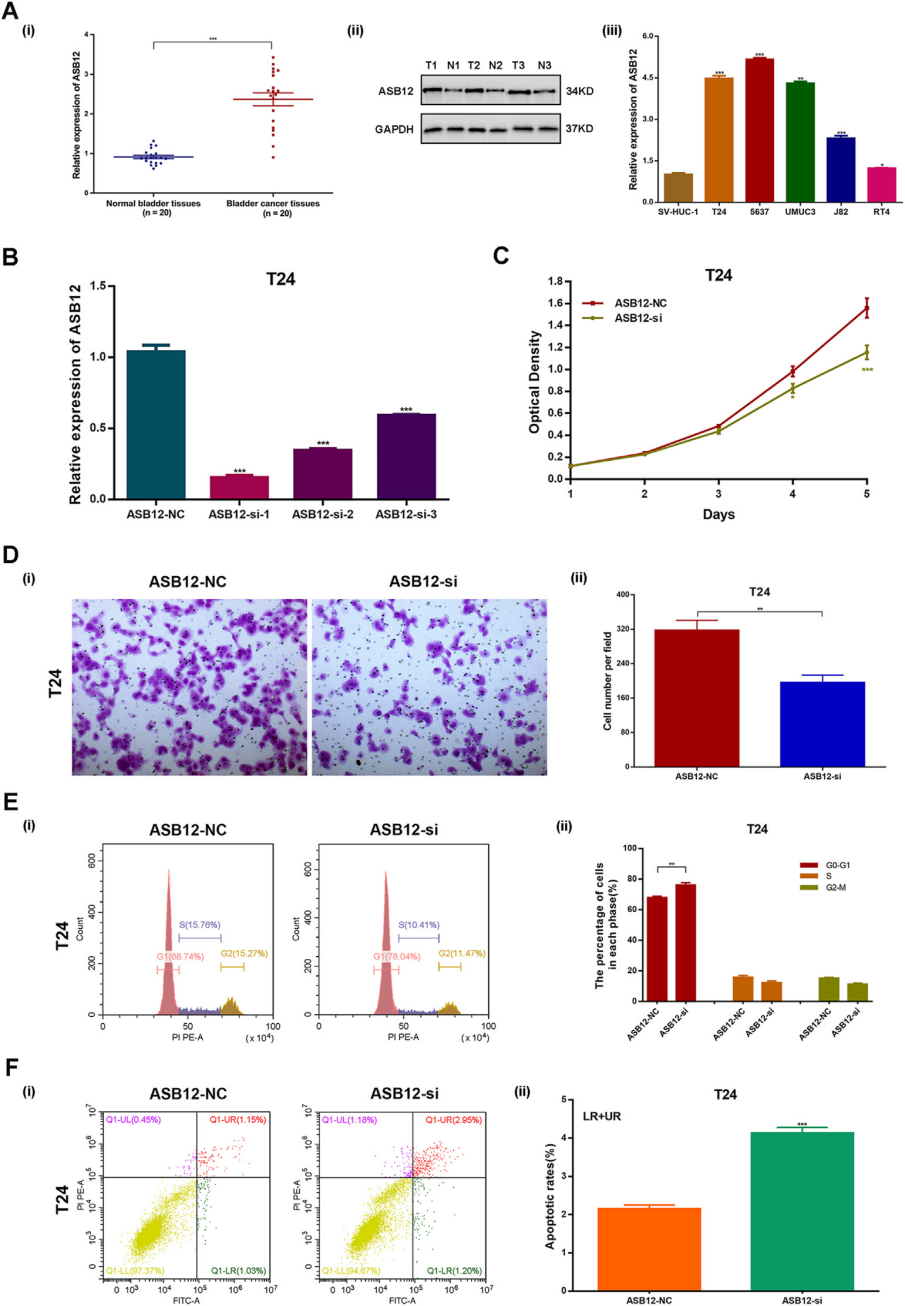
Down-regulation of ASB12 decreased proliferation and migration and increased cell cycle arrest and apoptosis in bladder cancer (BCa) cells. **(A)** ASB12 mRNA was highly expressed in tumor tissues of BCa patients (i), as well as ASB12 protein (ii), and ASB12 mRNA was highly expressed in BCa cell lines (iii). **(B)** Verification of ASB12 siRNA silencing efficacy in T24 cells by quantitative reverse transcription PCR **(C)** MTT assays indicated that ASB12 silencing decreased the proliferation capacity of T24 cells. **(D)** Migration assays showed that ASB12 silencing attenuated cell migration ability. **(E)** ASB12 silencing increased cell cycle arrest at the G0/G1 phase. **(F)** ASB12 silencing increased cell apoptosis. ∗*p* < 0.05, ∗∗*p* < 0.01, ∗∗∗*p* < 0.001.

As expected, ASB12 mRNA was highly expressed in tumor tissues (cohort 4) of BCa patients, as well as in BCa cell lines. ASB12 protein was highly expressed in tissues (cohort 4) of BCa patients (Fig. 6A). T24 cells were selected to test the function of ASB12. After silencing ASB12, MTT assays demonstrated that the down-regulation of ASB12 could repress BCa cell proliferation (Fig. 6B, C). Migration experiments also showed an obvious reduction in the *ASB12-si* group (Fig. 6D). Similar to lnc-TAF12–2:1, increased cell cycle arrest at the G0/G1 phase (Fig. 6E) and increased cell apoptosis (Fig. 6F) were observed after down-regulation of ASB12.

### Exosomal lnc-TAF12–2:1 facilitated BCa tumor growth *in vivo*

**Figure 7:**
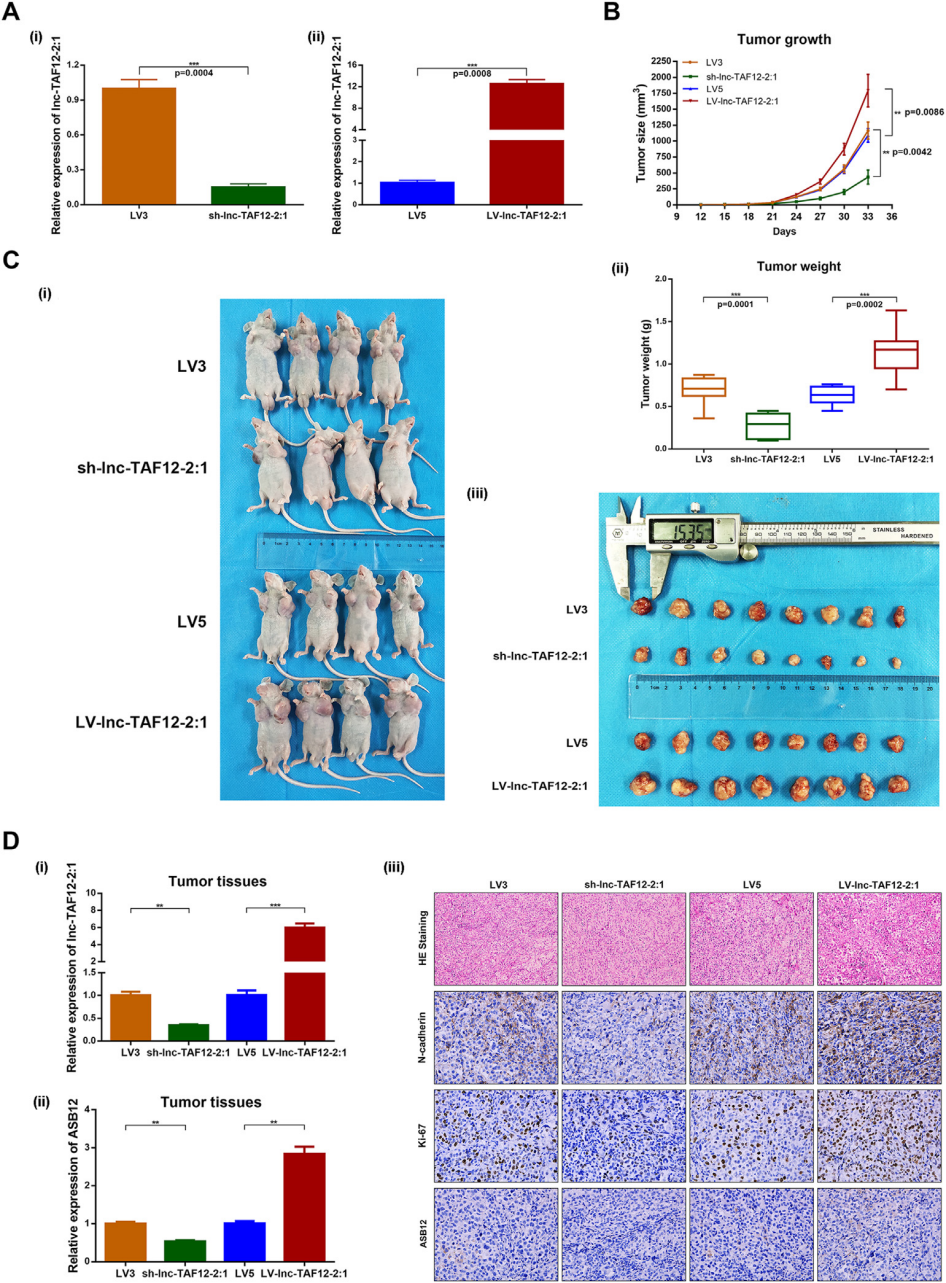
lnc-TAF12–2:1 promoted tumor growth *in vivo*. **(A)** The efficiency of sh-lnc-TAF12–2:1 (i) and lnc-TAF12–2:1 overexpression (LV-lnc-TAF12–2:1) (ii) lentivirus was confirmed at the mRNA level by quantitative reverse transcription PCR. **(B, C)** The continuous measurement of tumor growth activity and weight after injecting exosomes of sh-lnc-TAF12–2:1/LV-lnc-TAF12–2:1 or controls. **(D)** Detection of lnc-TAF12–2:1 and ASB12 expression in tumor tissues from mice (i), hematoxylin-eosin staining analyses of tumor tissues. Immunofluorescence analysis was used to detect the expression of N-cadherin, Ki-67, and ASB12 in mouse tumor tissues after treatment with sh-lnc-TAF12–2:1/LV-lnc-TAF12–2:1 and controls (ii).

Xenograft mouse models were established by subcutaneously injecting T24 cells infected with *sh-lnc-TAF12-2:1* or lnc-TAF12–2:1 overexpression (LV-lnc-TAF12–2:1) lentivirus to explore the pro-tumorigenic efficacy of lnc-TAF12–2:1 *in vivo*. The efficiencies of lnc-TAF12–2:1 knockdown and overexpression were initially evaluated in T24 cells (Fig. 7A). lnc-TAF12–2:1 silencing resulted in a significant reduction in mouse growth, whereas its overexpression caused tumor development *in vivo* compared with the control group (Fig. 7B). The measurement of tumor average weight and diameter also reported the same outcomes (Fig. 7C). The tumors were dissected to confirm the expression of lnc-TAF12–2:1 again. Hematoxylin-eosin staining and immunohistochemistry staining of tumors indicated that lnc-TAF12–2:1 knockdown alleviated the levels of ASB12, N-cadherin, and Ki-67, while lnc-TAF12–2:1 overexpression presented the opposite results (Fig. 7D).

**Figure 8:**
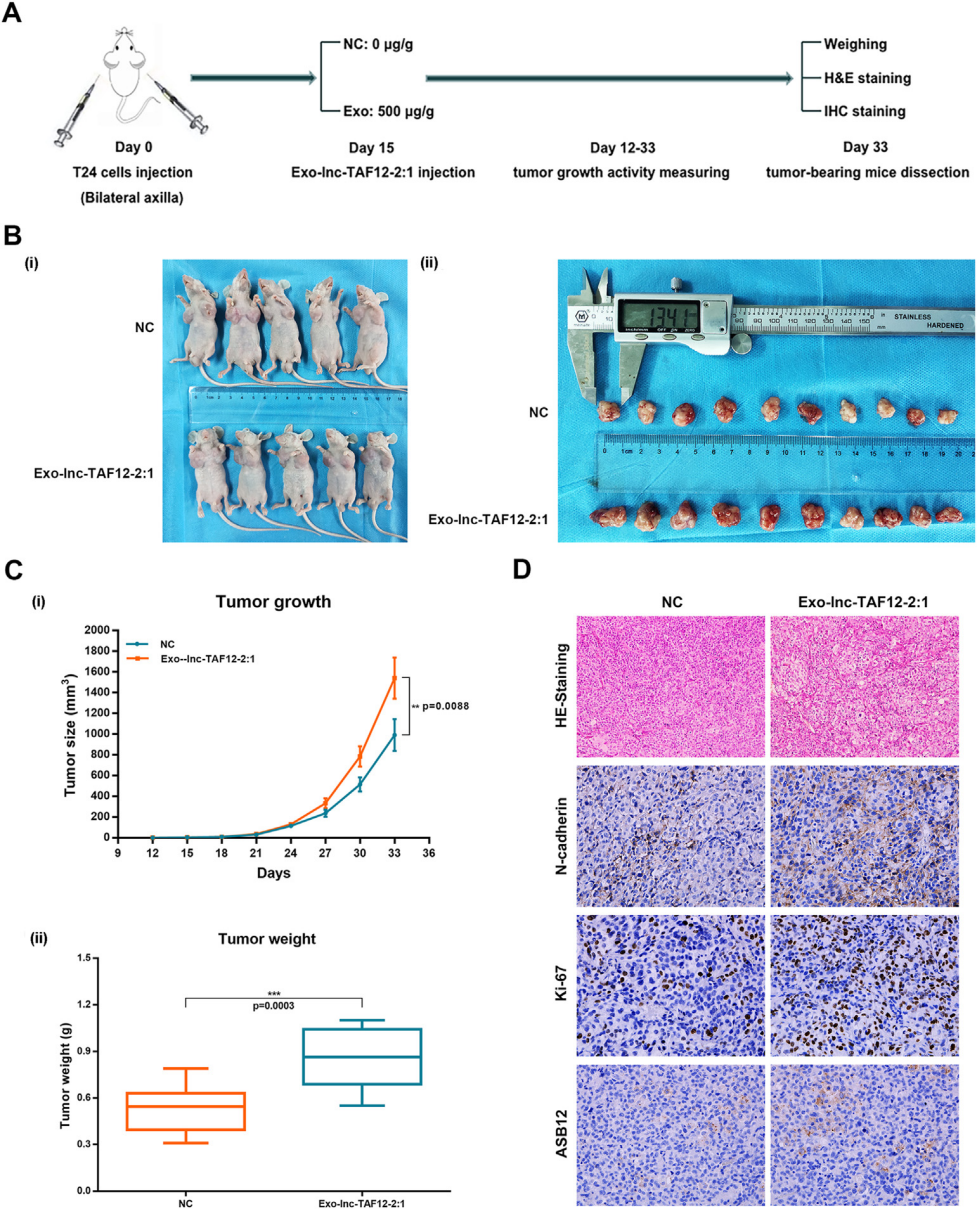
Tumor growth facilitation of exosomal lnc-TAF12–2:1 *in vivo*. **(A)** Flow chart of the xenograft mouse assay. **(B, C)** The continuous measurement of tumor growth activity and average weight after injecting exo-lnc-TAF12–2:1. **(D)** Hematoxylin-eosin staining analyses of tumor tissues after dissection. Immunofluorescence analysis was used to measure the expression of N-cadherin, Ki-67, and ASB12 in animal tumor tissues after extra exosomal lnc-TAF12–2:1 treatment.

As lnc-TAF12–2:1 displayed remarkable pro-tumorigenic effects *in vitro*, we began to assess the tumorigenic efficacy of exosomal lnc-TAF12–2:1 *in vivo*. First, T24 cells were subcutaneously injected into mice to construct xenograft mouse models (*n* = 5 per group). Fifteen days later, 500 μg/g exosomal lnc-TAF12–2:1 isolated from T24 cells or the same volume of saline solution was injected into nude mice, and the flow chart could be found in Figure 8A Tumor growth curves and average weight showed that exosomal lnc-TAF12–2:1 could facilitate BCa growth *in vivo* (Fig. 8B, C). The levels of ASB12, N-cadherin, and Ki-67 were also increased in the exosome lnc-TAF12–2:1 group compared with the NC group (Fig. 8D), which was consistent with the previous experimental results.

## Discussion

Numerous studies have emphasized that lncRNAs play essential roles in the tumor development and progression of BCa and closely correlate with inferior clinical prognosis.^28^, ^29^, ^30^ In the present study, we identified lnc-TAF12–2:1 according to lncRNA chip sequencing technology. Capsuled in urinary exosomes and secreted by tumor cells, lnc-TAF12–2:1 was highly expressed in BCa patients and had a considerable diagnostic performance with an AUC of 0.854.

Loss-of-function and gain-of-function assays were used to investigate the oncogenic function of lnc-TAF12–2:1. The results showed that it could not only stimulate tumor cell proliferation and migration and decrease cell cycle G1/G1 arrest and apoptosis *in vitro* but also promote tumor growth *in vivo*. Intriguingly, we found that exosomal lnc-TAF12–2:1 could transfer intercellular and convey essential biological information, which meant that lnc-TAF12–2:1 exerted its function with the aid of exosomal intercellular transmission. Exosomes are very excellent carriers of cell-to-cell communication and have been proven to mediate signals between cells within the tumor microenvironment by transferring their cargos to recipient cells.^31^ For example, exosomes released from normal cells can be transferred into BCa cells to enrich PTENP1 expression and take effect.^27^ Liu et al found that CAF-derived exosomes packaged lncRNA LINC01614 to interact with ANXA2 and p65 and increase glutamine uptake in lung adenocarcinoma cells.^32^ Our data suggested that exosomal lnc-TAF12–2:1 had reliable potential in clinical diagnosis and treatment. Hence, we considered exosomal lnc-TAF12–2:1 as a diagnostic biomarker without invasion and a therapeutic target, which was consistent with previous exosome studies in BCa.^27^^,^^31^^,^^33^

Accumulating evidence has proven that lncRNAs act as ceRNAs to competitively sponge miRNAs and regulate carcinoma pathogenesis. Chen et al found that DANCR (differentiation antagonizing non-protein coding RNA) served as a ceRNA to modulate the miR-149/MSI2 axis in BCa.^34^ Miao and colleagues discovered that LINC00612 promoted the development and metastasis of BCa by sponging miR-590 to up-regulate PHF14.^35^ Our research recognized that lnc-TAF12–2:1 was predominantly oriented in the cytoplasm and tended to construct a ceRNA regulatory network. Bioinformatics analysis helped us to find target miRNAs and mRNAs with complementary 3′UTR sequences and accelerated the process of mechanistic investigation. The interaction of lnc-TAF12–2:1, miR-7847–3p, and ASB12 was further supported by the dual luciferase reporter assay, RIP, and RNA pulldown assays. As shown in the mechanism diagram in Figure S6, BCa cells secreted lnc-TAF12–2:1, which was packaged in exosomes for transmission and promoted the expression of ASB12 by acting as a ceRNA and competitively sponging miR-7847–3p to mediate the proliferation, migration, cell cycle arrest, and apoptosis of tumor cells, thereby promoting the development of BCa. We concluded that lnc-TAF12–2:1 and ASB12 were both oncogenes and involved in the malignant phenotype of BCa.

Although miR-7847 has been explored as a negative apoptosis regulator in the antiviral immune response of invertebrates^36^ and miR-7847–3p has been identified as one of the signatures to distinguish Prader–Willi syndrome with or without steatosis,^37^ there are still no definitive reports to confirm the roles of miR-7847–3p in carcinoma. In other words, our study first illustrated that miR-7847–3p could participate in the BCa regulation mechanism of lnc-TAF12–2:1.

With the structure of N-terminal ANK repeats and a C-terminal SOCS box, ASB12 is a member of the ankyrin (ANK) repeat and SOCS box-containing (ASB) family. Previous research has found that the ASB family plays a regulatory role in various tumors and other diseases. Nie et al found that Notch-induced ASB2 could promote the ubiquitination of Notch to control tumor cell proliferation and differentiation.^38^ The tumorigenic properties of ASB3 and ASB4 were revealed in hepatocellular carcinoma cells.^39^^,^^40^ ASB6 was reported to participate in the regulation of an insulin-signaling pathway.^41^ Hou et al found that ASB1 was involved in the positive regulation of inflammatory responses.^42^ ANK is necessary for substrate domain recognition, and the SOCS box is involved in mediating ubiquitination and proteolytic degradation.^43^ Meanwhile, the ASB family can assemble with Cul5-Rbx2 to form E3 ubiquitin ligases,^44^ which are thought to be closely related to BCa progression in recent studies.^45^^,^^46^ The most dramatic feature of the ASB family is that they can drive compartment size, which has a greater probability of tumor development.^47^ However, there are still no studies concentrating on determining the role of ASB12 in malignancy. Our study revealed that ASB12 could promote tumor cell proliferation and migration and reduce cell cycle arrest as well as cell apoptosis in BCa cells. We believe that ASB12 could be considered a potential biomarker for future research.

## Conclusions

In summary, our data demonstrated that lnc-TAF12–2:1 was markedly up-regulated in urinary exosomes and tumor tissues of BCa patients and BCa cells. Down-regulation of exosomal lnc-TAF12–2:1 expression could repress BCa cell proliferation and migration and promote cell cycle arrest at the G0/G1 phase and cell apoptosis. Furthermore, exosomal lnc-TAF12–2:1 facilitated tumorigenesis by acting as a ceRNA to competitively sponge miR-7847–3p and prevented ASB12 from miRNA-mediated degradation. These results suggest that exosomal lnc-TAF12–2:1 is an unprecedented diagnostic and therapeutic biomarker that functions by influencing the miR-7847–3p/ASB12 regulatory axis in BCa.

## Ethics declaration

All animal experiments and use of clinical samples (such as urine and tissue specimens) and clinical information were approved by the Ethics Committee at Zhongnan Hospital of Wuhan University (No. 2021125), and all research procedures were compliant with the Helsinki Declaration. Written informed consent was obtained from the patient for publication of this case report and any accompanying images. A copy of the written consent is available for review.

## Author contributions

S.C., T.W., and X.W. conceived and designed this study. S.C., S.L., and T.W. collected clinical information and biosamples. S.C., J.C., S.L., and D.S. performed the analysis procedures. S.C. and T.W. analyzed the results. S.C., J.C., and X.W. contributed analysis tools. S.C., J.C., T.W., and X.W. contributed to the writing of the manuscript. X.W. supervised the research. All authors reviewed the manuscript.

## Funding

This study was funded by 10.13039/501100007046Wuhan University (No. 4206–413100049), the Science and Technology Department of Hubei Province Key Project (China) (No. 2022EJD001), Zhongnan Hospital of Wuhan University (No. ZNYQ2023002, KY0100000109), the Fundamental Research Funds for the Central Universities (China) (No. 2042022dx0003), Non-profit Central Research Institute Fund of Chinese Academy of Medical Sciences (No. 2020-PT320-004), and Young Elite Scientists Sponsorship Program by the China Association for Science and Technology (No. 2022QNRC001). The funders were not involved in the study design, data collection, analysis, publication decisions, or manuscript preparation.

## Data availability

The datasets used and/or analyzed during the current study are available from the corresponding authors upon reasonable request.

## Conflict of interests

The authors declared no conflict of interests.

## References

[1] Siegel R.L., Miller K.D., Wagle N.S., Jemal A. (2023). Cancer statistics, 2023. CA Cancer J Clin.

[2] Antoni S., Ferlay J., Soerjomataram I., Znaor A., Jemal A., Bray F. (2017). Bladder cancer incidence and mortality: a global overview and recent trends. Eur Urol.

[3] Patel V.G., Oh W.K., Galsky M.D. (2020). Treatment of muscle-invasive and advanced bladder cancer in 2020. CA Cancer J Clin.

[4] Catto J.W.F., Downing A., Mason S. (2021). Quality of life after bladder cancer: a cross-sectional survey of patient-reported outcomes. Eur Urol.

[5] Mitra A.P., Cai J., Miranda G. (2022). Management trends and outcomes of patients undergoing radical cystectomy for urothelial carcinoma of the bladder: evolution of the University of Southern California experience over 3347 cases. J Urol.

[6] Morillon YM 2nd, Su Z., Schlom J., Greiner J.W. (2019). Temporal changes within the (bladder) tumor microenvironment that accompany the therapeutic effects of the immunocytokine NHS-IL12. J Immunother Cancer.

[7] Lee Y.C., Lam H.M., Rosser C., Theodorescu D., Parks W.C., Chan K.S. (2022). The dynamic roles of the bladder tumour microenvironment. Nat Rev Urol.

[8] Walker J.M., O'Malley P., He M. (2022). Applications of exosomes in diagnosing muscle invasive bladder cancer. Pharmaceutics.

[9] Kalluri R., LeBleu V.S. (2020). The biology**,** function**,** and biomedical applications of exosomes. Science.

[10] Dai J., Su Y., Zhong S. (2020). Exosomes: Key players in cancer and potential therapeutic strategy. Signal Transduct Targeted Ther.

[11] Davidson S.M., Boulanger C.M., Aikawa E. (2023). Methods for the identification and characterization of extracellular vesicles in cardiovascular studies: from exosomes to microvesicles. Cardiovasc Res.

[12] Fan Y., Chen Z., Zhang M. (2022). Role of exosomes in the pathogenesis, diagnosis, and treatment of central nervous system diseases. J Transl Med.

[13] Fang Y., Ni J., Wang Y.S. (2023). Exosomes as biomarkers and therapeutic delivery for autoimmune diseases: opportunities and challenges. Autoimmun Rev.

[14] Isaac R., Reis F.C.G., Ying W., Olefsky J.M. (2021). Exosomes as mediators of intercellular crosstalk in metabolism. Cell Metabol.

[15] Paskeh M.D.A., Entezari M., Mirzaei S. (2022). Emerging role of exosomes in cancer progression and tumor microenvironment remodeling. J Hematol Oncol.

[16] Li P., Mi Q., Yan S. (2023). Characterization of circSCL38A1 as a novel oncogene in bladder cancer via targeting ILF3/TGF-β2 signaling axis. Cell Death Dis.

[17] Yang C., Wu S., Mou Z. (2022). Exosome-derived circTRPS1 promotes malignant phenotype and CD8^+^ T cell exhaustion in bladder cancer microenvironments. Mol Ther.

[18] Statello L., Guo C.J., Chen L.L., Huarte M. (2021). Gene regulation by long non-coding RNAs and its biological functions. Nat Rev Mol Cell Biol.

[19] Schmitt A.M., Chang H.Y. (2016). Long noncoding RNAs in cancer pathways. Cancer Cell.

[20] Xu Y., Wu W., Han Q. (2019). New insights into the interplay between non-coding RNAs and RNA-binding protein HnRNPK in regulating cellular functions. Cells.

[21] Bridges M.C., Daulagala A.C., Kourtidis A. (2021). LNCcation: lncRNA localization and function. J Cell Biol.

[22] Zhang Y., Luo M., Cui X., O'Connell D., Yang Y. (2022). Long noncoding RNA NEAT1 promotes ferroptosis by modulating the miR-362-3p/MIOX axis as a ceRNA. Cell Death Differ.

[23] Zhong C., Yu Q., Peng Y. (2021). Novel LncRNA OXCT1-AS1 indicates poor prognosis and contributes to tumorigenesis by regulating miR-195/CDC25A axis in glioblastoma. J Exp Clin Cancer Res.

[24] Huang X., Yuan T., Tschannen M. (2013). Characterization of human plasma-derived exosomal RNAs by deep sequencing. BMC Genom.

[25] Wang Y., Zhang M., Zhou F. (2020). Biological functions and clinical applications of exosomal long non-coding RNAs in cancer. J Cell Mol Med.

[26] Chen C., Luo Y., He W. (2020). Exosomal long noncoding RNA LNMAT2 promotes lymphatic metastasis in bladder cancer. J Clin Invest.

[27] Zheng R., Du M., Wang X. (2018). Exosome-transmitted long non-coding RNA PTENP1 suppresses bladder cancer progression. Mol Cancer.

[28] He W., Zhong G., Jiang N. (2022). Long noncoding RNA BLACAT2 promotes bladder cancer-associated lymphangiogenesis and lymphatic metastasis. J Clin Invest.

[29] Cai H., Xu H., Lu H. (2022). lncRNA SNHG1 facilitates tumor proliferation and represses apoptosis by regulating PPARγ ubiquitination in bladder cancer. Cancers.

[30] Liu P., Fan B., Othmane B. (2022). m^6^A-induced lncDBET promotes the malignant progression of bladder cancer through FABP5-mediated lipid metabolism. Theranostics.

[31] Akoto T., Saini S. (2021). Role of exosomes in prostate cancer metastasis. Int J Mol Sci.

[32] Liu T., Han C., Fang P. (2022). Cancer-associated fibroblast-specific lncRNA LINC01614 enhances glutamine uptake in lung adenocarcinoma. J Hematol Oncol.

[33] Geng H., Zhou Q., Guo W. (2021). Exosomes in bladder cancer: novel biomarkers and targets. J Zhejiang Univ - Sci B.

[34] Chen Z., Chen X., Xie R. (2019). DANCR promotes metastasis and proliferation in bladder cancer cells by enhancing IL-11-STAT3 signaling and CCND1 expression. Mol Ther.

[35] Miao L., Liu H.Y., Zhou C., He X. (2019). LINC00612 enhances the proliferation and invasion ability of bladder cancer cells as ceRNA by sponging miR-590 to elevate expression of PHF_14_. J Exp Clin Cancer Res.

[36] Gong Y., Kong T., Ren X. (2020). Exosome-mediated apoptosis pathway during WSSV infection in crustacean mud crab. PLoS Pathog.

[37] Pascut D., Tamini S., Bresolin S. (2018). Differences in circulating microRNA signature in Prader-Willi syndrome and non-syndromic obesity. Endocr Connect.

[38] Nie L., Zhao Y., Wu W., Yang Y.Z., Wang H.C., Sun X.H. (2011). Notch-induced Asb2 expression promotes protein ubiquitination by forming non-canonical E3 ligase complexes. Cell Res.

[39] Zhang W., Liu F., Che Z. (2019). ASB3 knockdown promotes mitochondrial apoptosis via activating the interdependent cleavage of Beclin 1 and caspase-8 in hepatocellular carcinoma. Sci China Life Sci.

[40] Au V., Tsang F.H., Man K., Fan S.T., Poon R.T.P., Lee N.P. (2014). Expression of ankyrin repeat and SOCS box containing 4 (ASB4) confers migration and invasion properties of hepatocellular carcinoma cells. Biosci Trends.

[41] Wilcox A., Katsanakis K.D., Bheda F., Pillay T.S. (2004). Asb6, an adipocyte-specific ankyrin and SOCS box protein, interacts with APS to enable recruitment of elongins B and C to the insulin receptor signaling complex. J Biol Chem.

[42] Hou P., Jia P., Yang K. (2021). An unconventional role of an ASB family protein in NF-κB activation and inflammatory response during microbial infection and colitis. Proc Natl Acad Sci USA.

[43] Kile B.T., Schulman B.A., Alexander W.S., Nicola N.A., Martin H.M.E., Hilton D.J. (2002). The SOCS box: a tale of destruction and degradation. Trends Biochem Sci.

[44] Kohroki J., Nishiyama T., Nakamura T., Masuho Y. (2005). ASB proteins interact with Cullin 5 and Rbx2 to form E3 ubiquitin ligase complexes. FEBS Lett.

[45] Xu H., Ju L., Xiong Y. (2021). E3 ubiquitin ligase RNF126 affects bladder cancer progression through regulation of PTEN stability. Cell Death Dis.

[46] Jing W., Wang G., Cui Z. (2022). FGFR3 destabilizes PD-L1 via NEDD4 to control T-cell-mediated bladder cancer immune surveillance. Cancer Res.

[47] Liu P., Verhaar A.P., Peppelenbosch M.P. (2019). Signaling size: ankyrin and SOCS box-containing ASB E3 ligases in action. Trends Biochem Sci.

