# Supplementary material for: Urinary exosomal lnc-TAF12–2:1 promotes bladder cancer progression through the miR-7847–3p/ASB12 regulatory axis
Source: Genes Dis. 2024 Aug 5;12(4):101384. doi: 10.1016/j.gendis.2024.101384 (PMC12036056; doi:10.1016/j.gendis.2024.101384)
Supplement: Multimedia component 1 [file mmc1.docx]

**Supplementary Figures**

**
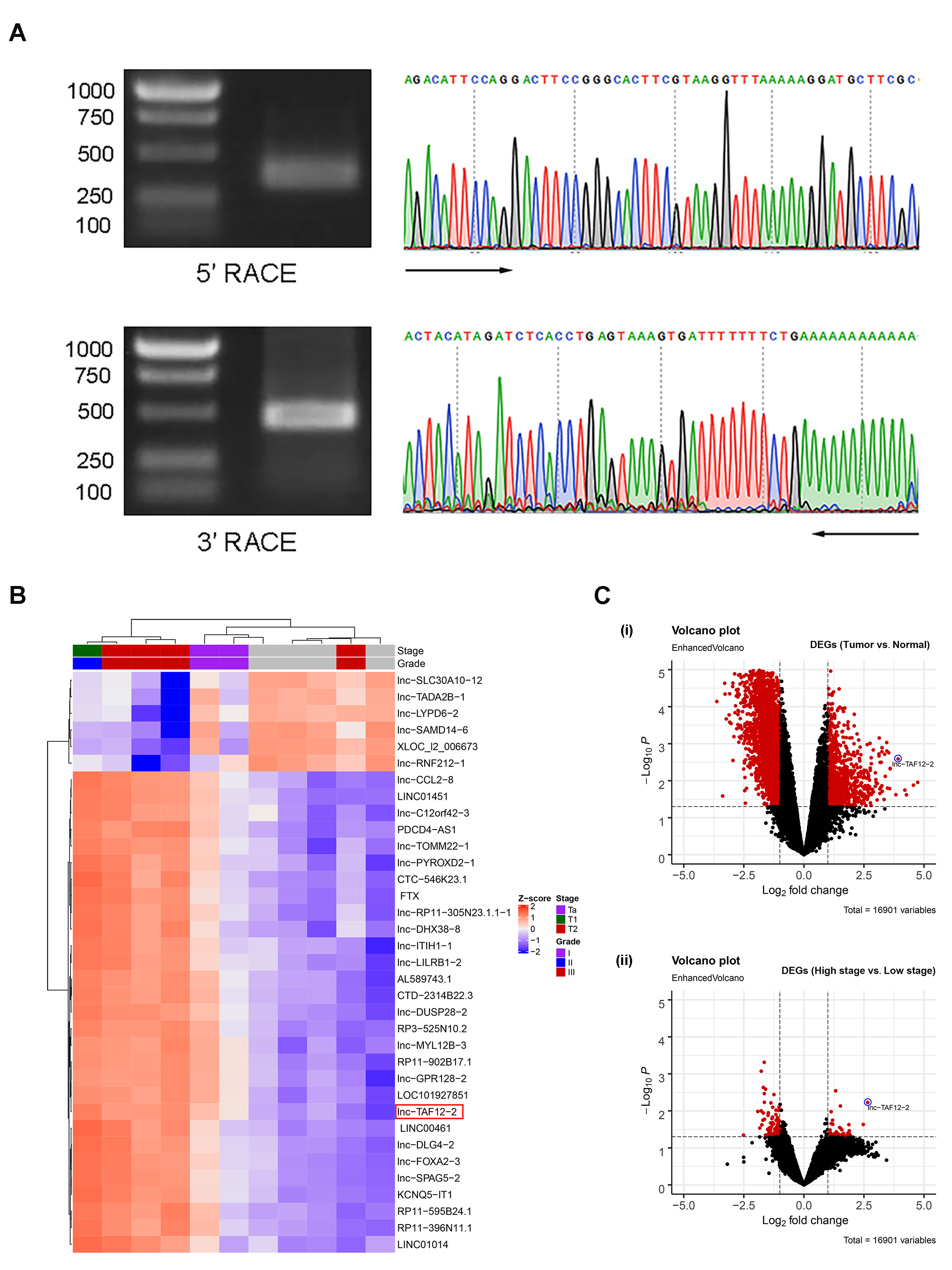
**

**Figure S1. Identification of a novel lnc-TAF12-2:1, which was highly expressed in BCa patients. (A)** 5′ and 3′ rapid amplification of cDNA ends (RACE) assays in Bladder cancer tissues to detect the whole sequence of lnc-TAF12-2:1. Left; a gel electrophoresis image of PCR products from the 5′-RACE and 3′-RACE assays. Right; sequencing of PCR products indicated the boundary between the universal anchor primer and lnc-TAF12-2:1 sequences. **(B)** Heat map showed differentially expression lncRNAs in urinary exosomes between BCa patients and normal health controls, lnc-TAF12-2:1 was upregulated in BCa patients. **(C)** Volcano plot presented differentially expression lncRNAs in urinary exosomes between tumor vs. nomal and high-stage tumor vs. low-stage tumor.

**
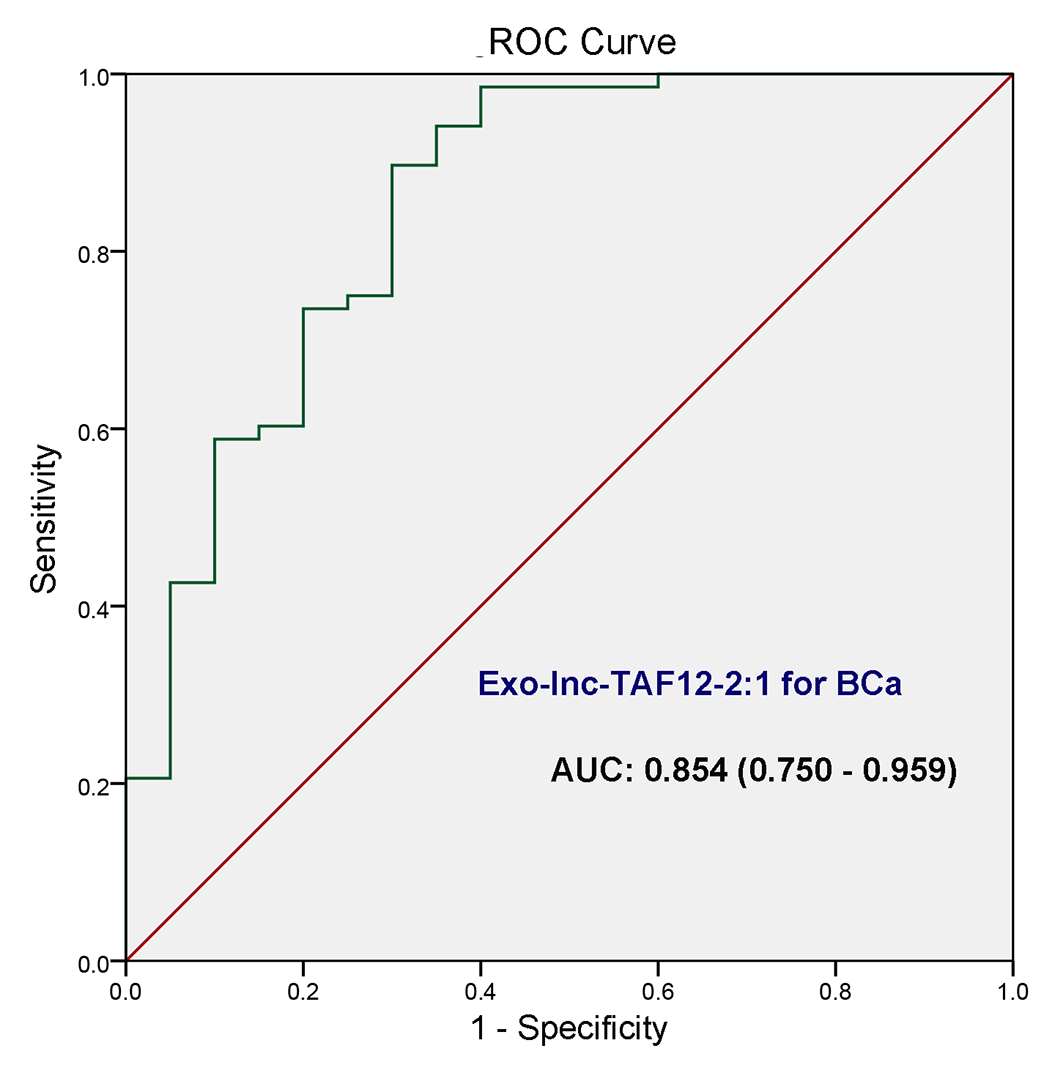
**

**Figure S2. ROC curve for diagnosis of BCa patients utilizing exosomes lnc-TAF12-2:1.** The area under the curve of lnc-TAF12-2:1 for diagnosing BCa was 0.854 (95% CI = 0.750 - 0.959).

**
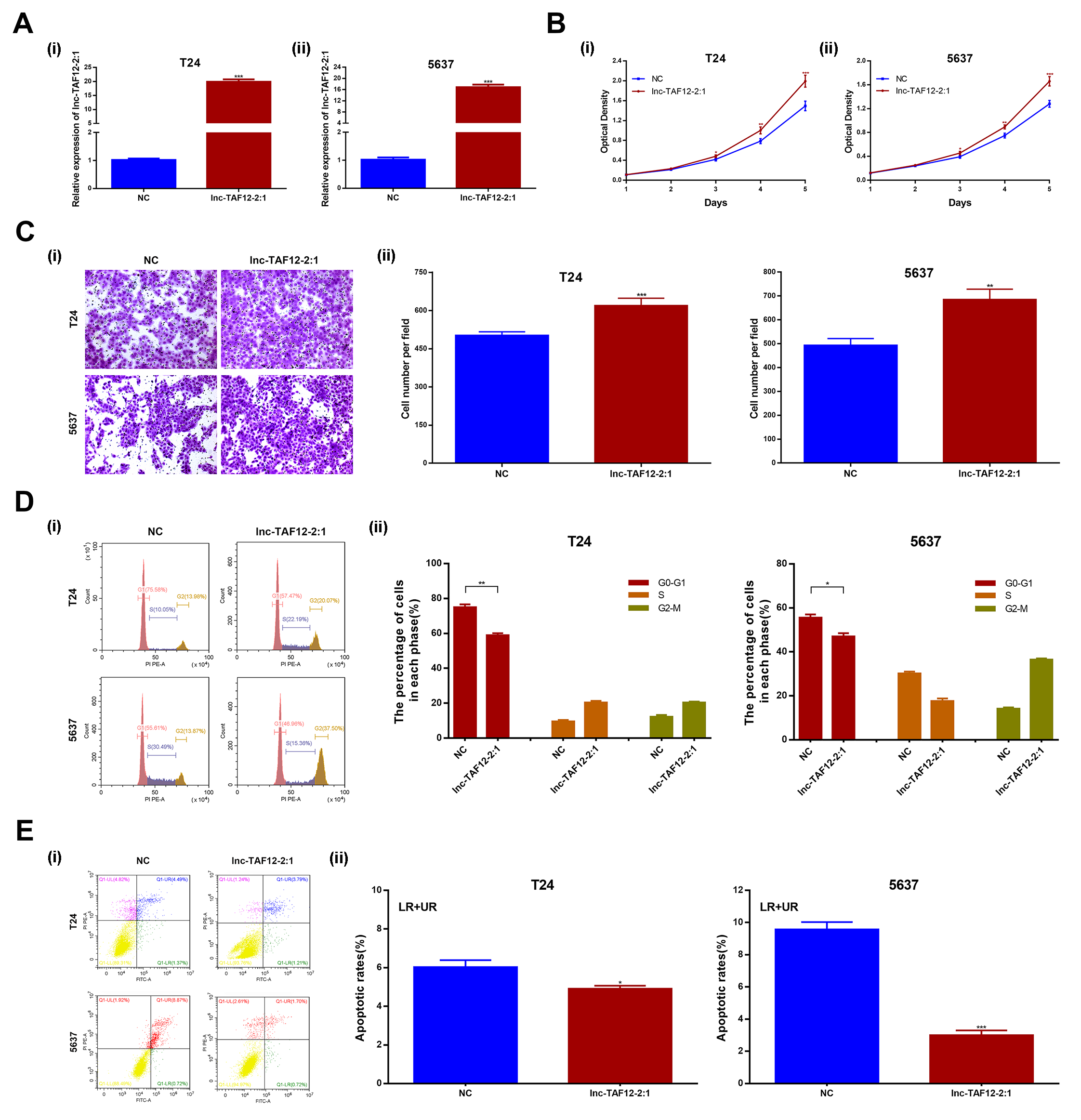
**

**Figure S3. Upregulation of lnc-TAF12-2:1 expression promoted BCa cell proliferation, migration and reduced cell cycle arrest and apoptosis. (A)** Verification of lnc-TAF12-2:1 overexpression efficacy at the mRNA level in T24 (i) and 5637 (ii) cells by qRT-PCR. **(B)** MTT assays indicated that lnc-TAF12-2:1 overexpression increased the capacity of proliferation in T24 (i) and 5637 (ii) cells. **(C)** Migration assays depicted that lnc-TAF12-2:1 overexpression enhanced cell migration ability. **(D)** lnc-TAF12-2:1 overexpression reduced cell cycle arrest at the G0/G1 phase. **(E)** lnc-TAF12-2:1 overexpression decreased cell apoptosis. * *p* < 0.05; ** *p* < 0.01; *** *p* < 0.001.

**
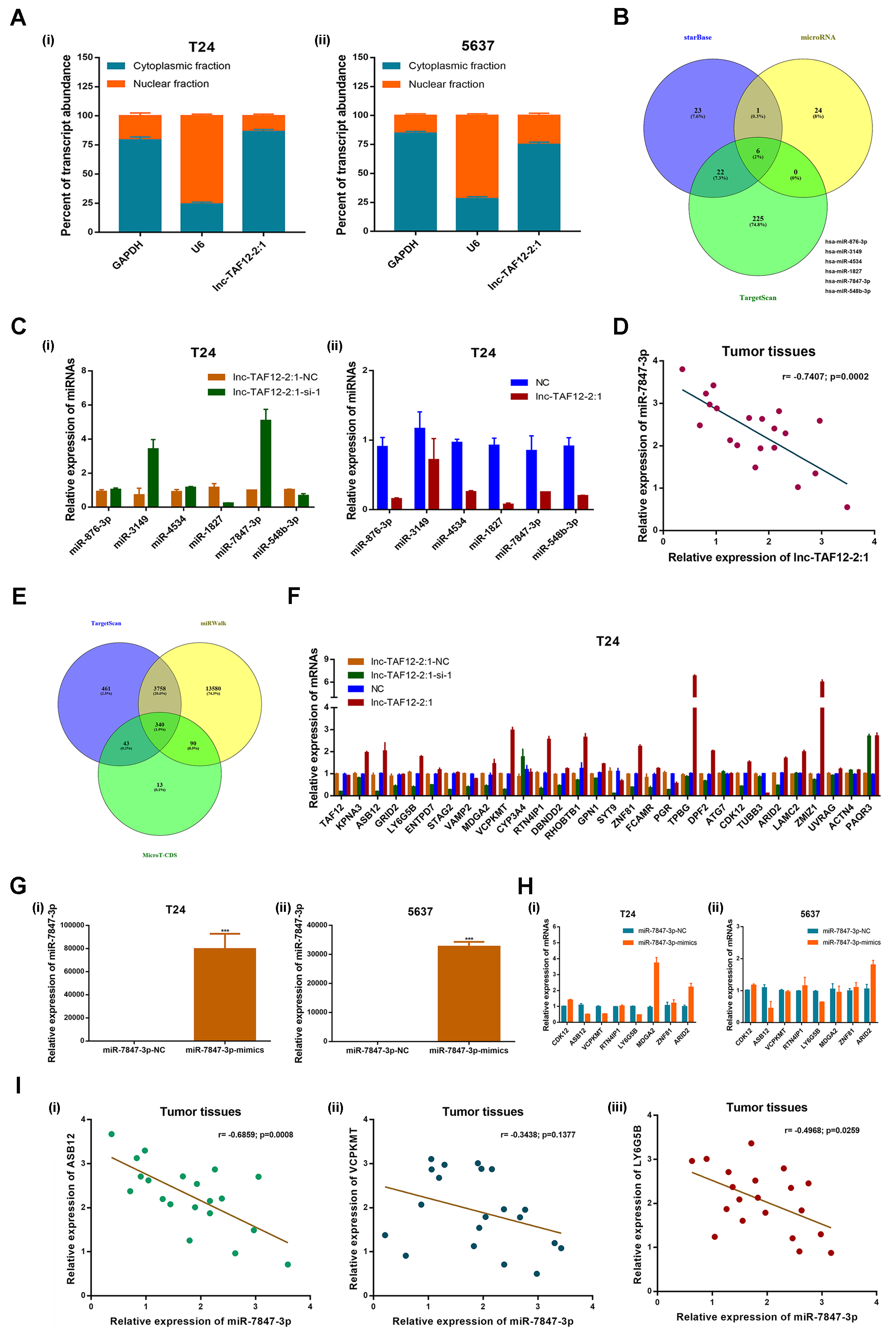
**

**Figure S4. Predicting target miRNAs and downstream mRNAs of lnc-TAF12-2:1. (A)** lnc-TAF12-2:1 expression level detection in nuclear and cytosolic fractions of T24 (i) and 5637 (ii) cells. **(B)** Databases of starBase, microRNA, TargetScan were used to predict possible target miRNA of lnc-TAF12-2:1. **(C)** Expression changes in miRNA after downregulation and upregulation of lnc-TAF12-2:1. **(D)** The correlation between the expression level of lnc-TAF12-2:1 and miR-7847-3p in 20 BCa patients analyzed by linear regression. **(E)** TargetScan, miRWalk, MicroT-CDS were exploited to predict downstream mRNA of miR-7847-3p. **(F)** Expression changes in top 20 mRNAs after downregulation and upregulation of lnc-TAF12-2:1. **(G)** The overexpression transfection efficiency of miR-7847-3p mimics. **(H)** Relative quantification of mRNA after transfecting miR-7847-3p mimics to search for mRNAs contrast to the expression of miR-7847-3p. **(I)** The correlation between miR-7847-3p and ASB12, VCPKMT, LY6G5B analyzed by linear regression.

**
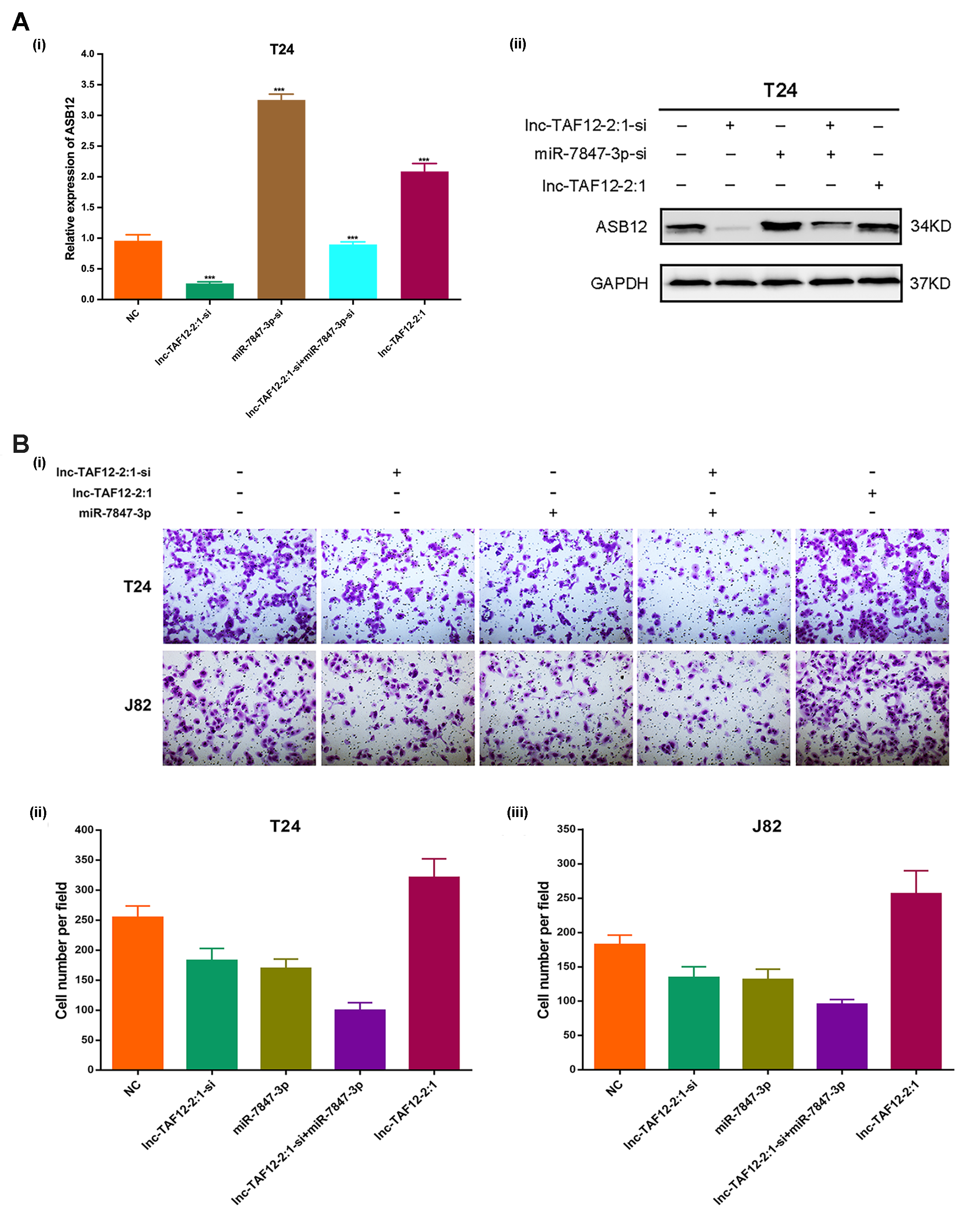
**

**Figure S5. Rescue experiments. (A)** lnc-TAF12-2:1 could regulate the expression of ASB12 by interacting with miR-7847-3p. **(B)** miR-7847-3p overexpression increased the migration inhibition induced by silencing lnc-TAF12-2:1 in BCa cells in T24 and J82 cells.

**
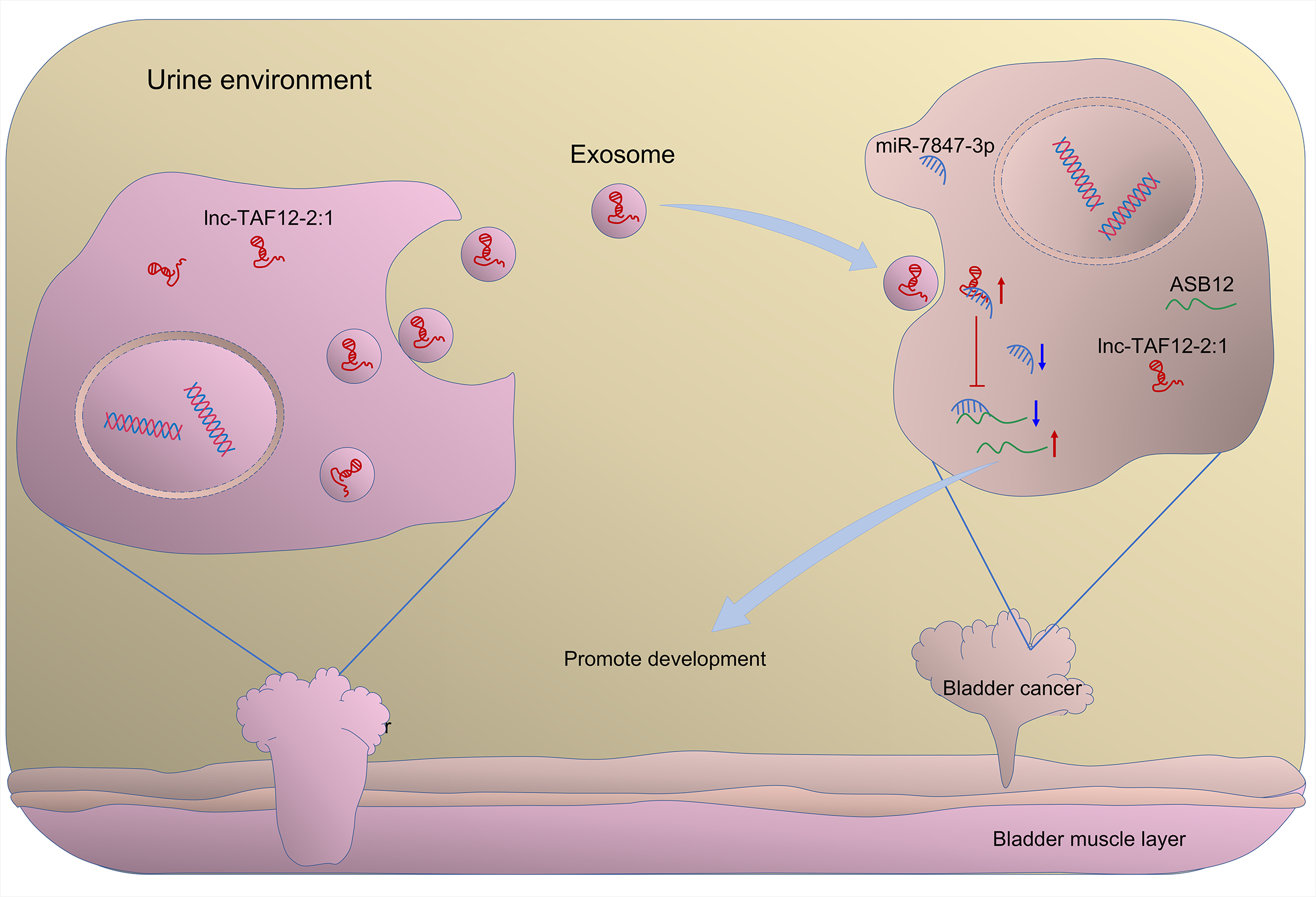
**

**Figure S6. Mechanistic diagram of exosomal lnc-TAF12-2:1 regulating the miR-7847-3p/ASB12 axis to promote the development of BCa.** The exosomal lnc-TAF12-2 in the cytoplasm of BCa might act as a ceRNA, competitively sponge with miR-7847-3p to release the inhibitory effect of ASB12 and upregulate the expression of ASB12 indirectly, thereby promoting the occurrence and development of BCa.
