# Supplementary material for: Urinary exosomal lnc-TAF12–2:1 promotes bladder cancer progression through the miR-7847–3p/ASB12 regulatory axis
Source: Genes Dis. 2024 Aug 5;12(4):101384. doi: 10.1016/j.gendis.2024.101384 (PMC12036056; doi:10.1016/j.gendis.2024.101384)
Supplement: Multimedia component 2 [file mmc2.docx]

**Supplementary material and methods**

**Full-length lnc-TAF12-2:1**

lnc-TAF12-2:1 (ENSG00000233427): AGACATTCCAGGACTTCCGGGCACTTC

GTAAGGTTTAAAAAGGATGCTTCGCGTTTTCTCTCTCCTTTTTGGAGACAGATTCGCAGTGGTCGCTTCTTCTCCTTGGATTTGTTAAGGATTCCAAGTAACTCTTATTTGGAGAGAAGACGATCTGCACTTCGCATTTTGGCATTGACATTTAATTTTAGGGTCCTTTATATAGAAGGGAGAGTAGGTAAACTGATTTTTTTTTTTAACAGGGAGGGTTTGACAATCTTTGGCAGACTTGGAGCAAAAGATTGAGGTGCATTTCATGCCTCCTTTTGAGAGTCTTGCTCTGTCGCCCAGGCTGTAGTGCAGTGGCGCAATCTTGGCTGCAACCTCAGCCTCCCAAGTAGCTGGGATTACAAACATAAGCCACCACGCCCAGCCCTCATACCTCTTTTAAAAGTCGACCTGTTTTGCAGAAAGTCTGCTGTTTTTGTACTAAAGGCTTTGGAATTTGGCATTTAGCTAGGAATGCACATTCTTTCACCTCATTCATACTTTAAGAACCACAGAAGTGACTCTGCTTGGCCAGAAGGCACACTGTGTTGGTGGTTATATTAAAAGTCCTTGAGTATTTTGCTTTTCATGATCTTGCTCACTGCAACTTCCGCCTCCCAGGTTCAGGCGATTCTCCTGCCTCAGCCTCCCAAGTAGCTGCGACTACAGGCGTGTAGCACCACACCTGGCTAATTTTTGTATTTTTAGTAGAGATGAGGTTTCACCATATTGGCCAGGCTGTTCTCAACTCCTGACCTCGTGATCCGCCCACCTCAGCCTCCTAAAGTGCTGGGATTACAGCTGTGAGCCACCCTGCCCGGCCACTTTTGTATGATTTCTAATGTATTTGTAATTTACCTAACAAATTGCCTAATCTGCTATGTTAATGTATTTATGAATTAAAATAAATACGACTGCATGTTTGTGGTTCATTTTTGTGGAGGTGGCTGTGGTGACATCAGCCAAGAATCTGAATGGTACTGTTGAAGGAAACTAGCATGATAGCTTCAGTTCTAAAGGCCCTGAAACCTAGTCTCAGGTGGGTCCCCCTTGGGTTCACTTTATATTGGCAGTTTATTGGGAAAATGGATATTAGGTCCTGACCAATAGGACCGTAAGTCTGGGTTGAGTGCAAGATGAGTTAGACCGATTCTTTAGCTTCCTGCAGTGTAGTGGAGGAAAAATCGATGGTAGCAACGGGAGGTTGTATCCCTAGCTGATGAGTTGTATGAGCCTCTACTACCTGGCGCACCTCCGCCTGAAGATTGCCAGAATTGCTTGCCTCATGACGTGAGTCACAATGGAAACTTTGTCAAGCCCCCTGCACTGGCTGCCAACATAAATGTTCAGTACCCTGAAGGATGGGACTGAAGGGGGATCATCTAGAAGGTAAAGTTACCTACTGGCATAGGGGAGGTGGGACAGCCGTTAAGCCATTTGGAACTTGATGGAGACAGGTTTGAGGGAGGTGGGTGAGATTGGAGTTTGGTGGACTGTAGAGCTTGCTTGCCAAGGTGTTGAGGTCAGGGTTGGTTTGAGAATGGAAGCTAGTTACTAGCTATGATTGTGGGGGAACACAGCTTGATTTTTCTTACAAGCTAAGAGGAGTGAGGCAGTGTTTAAGAGGGCATGTTAAATGCAGCCAGGCTTGGTGGCTCACACCCGTAATCCCAGCACTTAGGCTAAGGCAGGCGGATCACAACATCTAGAGATCCTGGCCAACGCGGTGAAACCCTGTCTGTACTAAAAATACAAAATAACTGGGCATGGTGGTGTGCACCTGTGGGAGGCTGAGGCAGAATTGCTGGAACCCGGGAGATGGAGGTTGTACTGAGCTGAGACCTTGCCACTGCGCTCCAGCCTGGTGACAGAGTTAAGTCTCAAAAAAAAGGCATCTTCCTAAAGCAATTGTATTTGTGCTTACCTGTGCCAGGCACTGTTCTAGGTAAGCACTAAGTGGGCTTTAATACAGCATATTCCAATGGGGAATCCCAGGAACCAAAAGACTAATTGTCCAAGTCCACAACTAGAAGTGGCACCTCTGCAGAAACAAGCATCAAATTCCCTGCTCAGGAAGAAGCCAGATGAGTCAGCCCCATTCGTCTGTATGCCCAGTCCCATCCGTGTCCTGCTGTAACTACATAGATCTCACCTGAGTAAAGTGATTTTTTTCTGAAAAAAAAAAAA.

**5' and 3′ rapid amplification of cDNA ends (RACE) analysis**

Total RNA was isolated using TRIzol Plus RNA Purifica tion Kit (Invitrogen), and reverse transcription synthesis of cDNA using PrimeScript 1st Strand cDNA Synthesis Kit (Takara), according to the manufacturer’s in structions. 5′ RACE and 3′ RACE were performed using SMARTer® RACE 5’/3’Kit (Clontech) according to the manufac turer’s instructions. The following gene-specific primers (GSP) are used for PCR: 5′-GCCAAGCAGAGTCACTTCTGTGGTTC-3′ (5′ RACE GSP1), 5′-GATTACGCCAAGCTtGCCAAGATTGCGCCACTGCACTAC-3′ (5′ RACE GSP2), 5′-GGCAGGCGGATCACAACATC-3′ (3′ RACE GSP1), 5′-GGGATCCTCTAGAGATtGAGGCTGAGGCAGAATTGCT-3′ (3′ RACE GSP2).

**Cell proliferation assay**

An MTT assay was performed to determine cell proliferation. BCa cells were transfected, plated into 96-well plates in 200 μl of medium, cultured for 5 days, added to 20 μl of MTT solution (5 mg/ml) in each well, and incubated for 4 h. A microplate reader (Cat. #SpectraMax M2, Molecular Devices, USA) was measured at 490 m.

**Clonogenic assay**

In the clonogenic assay, the transfected BCa cells were plated in 6-well plates and cultured for 2 weeks (1000 cells per well). The cells were fixed with 4% paraformaldehyde for 30 mins after washing gently twice with PBS and then stained with crystal violet. The colony number was calculated.

**Migration assay**

The transfected BCa cells were plated in 200 μl serum-free medium in the upper Transwell chamber (Corning, USA). Then, 600 μl of medium containing 10% FBS was added to the lower chamber. After culturing for 24 h, we removed the cells on the upper chamber. The cells in the lower chamber were fixed with 4% paraformaldehyde and stained with crystal violet. The number of stained cells was calculated under an inverted phase contrast microscope and photographed in 3 random fields.

**Flow cytometry analyses for cell cycle and cell apoptosis**

The transfected BCa cells were collected, centrifuged and washed twice with cold PBS. For the cell cycle, the cell precipitate was resuspended in a 1× DNA staining solution containing propidium iodide and permeabilization solution (Multi Sciences, China) in the dark and incubated at 37°C for 30 min. For cell apoptosis, cells were collected and stained with an annexin V-fluorescence isothiocyanate (FITC)/PI Apoptosis Detection Kit (BD Biosciences, USA). Flow cytometry (Cat. #FC500, Beckman, USA) was used to analyse the cell cycle distribution and cell apoptosis.

**Hematoxylin and eosin (H&E) staining**

The mouse tumor tissues were stained with hematoxylin & eosin. We deparaffinized and continuously rehydrated the sections with xylene, 100% ethanol, 96% ethanol, 80% ethanol, 70% ethanol and H_2_O and then stained the sections with 10% hematoxylin (Sigma‒Aldrich). The cell nuclei appeared after washing. One percent eosin (Sigma‒Aldrich) containing 0.2% glacial acetic acid was used to stain the cytoplasm. The slides were quickly washed and dehydrated in 70%, 80%, 96%, 100% ethanol and xylene. We used an inverted phase contrast microscope (Cat. #DMI 1, Leica, Wetzlar, Germany) to photograph the sections.

**Immunofluorescence staining**

Immunofluorescence staining of fixed BCa cells was accomplished by Biofavor Biotech Ltd. (Wuhan, China). ActinRed staining solution was added to the cell slide at a dilution of 1:600 and incubated at room temperature for 20 mins. Slides were rinsed with PBS three times, the tablets were sealed with DAPI, and stained slides were observed using confocal fluorescence microscopy (Nikon C2+ Confocal Microscope, Japan).

**Immunohistochemistry (IHC) staining**

For IHC staining, a proportion of surgical tissue specimens were fixed with formalin for paraffin embedding. IHC analyses were performed on 4 μm thick sections. Briefly, each slide was incubated with primary antibodies against ASB12, N-cadherin and Ki-67 overnight after a series of procedures (deparaffin, antigen retrieval, rinse). This was followed by incubation with the anti-rabbit IgG-HRP antibody for 30 min. The membrane was then washed five times with TBST and enriched with the brown color of DAB Enhancer (Dako, China). The expression of ASB12, N-cadherin and Ki-67 was evaluated by three experienced pathologists. We used a phase contrast microscope to analyse IHC sections.
