# Supplementary material for: Urinary exosomal lnc-TAF12–2:1 promotes bladder cancer progression through the miR-7847–3p/ASB12 regulatory axis
Source: Genes Dis. 2024 Aug 5;12(4):101384. doi: 10.1016/j.gendis.2024.101384 (PMC12036056; doi:10.1016/j.gendis.2024.101384)
Supplement: Multimedia component 3 [file mmc3.docx]

**Supplementary Tables**

**Supplementary Table S1. The clinicopathological information of Cohort 1.**

| **No.** | **Category** | **Gender** | **Age** | **Tumour stage** | **Tumour Grade** | **Infiltration** |
| --- | --- | --- | --- | --- | --- | --- |
| 1 | BCa patient | Male | 33 | Ta | G1 | NMIBC |
| 2 | BCa patient | Male | 68 | T2 | G3 | MIBC |
| 3 | BCa patient | Female | 55 | T1 | G1 | NMIBC |
| 4 | BCa patient | Female | 70 | Ta | G1 | NMIBC |
| 5 | BCa patient | Male | 71 | T1 | G2 | NMIBC |
| 6 | BCa patient | Male | 84 | T2 | G3 | MIBC |
| 7 | BCa patient | Male | 53 | T1 | G1 | NMIBC |
| 8 | BCa patient | Female | 82 | T2 | G3 | MIBC |
| 9 | Healthy volunteers | Male | 28 | / | / | / |
| 10 | Healthy volunteers | Male | 41 | / | / | / |
| 11 | Healthy volunteers | Female | 62 | / | / | / |
| 12 | Healthy volunteers | Female | 25 | / | / | / |

**Supplementary Table S2. The clinicopathological information of Cohort 2.**

| **Patient number** | **Gender** | **Age** | **Tumour stage** | **Tumour Grade** | **Infiltration** |
| --- | --- | --- | --- | --- | --- |
| 1 | Female | 68 | T2 | G2 | MIBC |
| 2 | Male | 64 | T1 | G1 | NMIBC |
| 3 | Male | 73 | T3 | G3 | MIBC |
| 4 | Female | 77 | T1 | G1 | NMIBC |
| 5 | Male | 58 | T2 | G3 | MIBC |

**Supplementary Table S3. The clinicopathological information of Cohort 3.**

| **No.** | **Category** | **Gender** | **Age** | **Tumour stage** | **Tumour Grade** | **Infiltration** |
| --- | --- | --- | --- | --- | --- | --- |
| 1 | Healthy volunteers | Male | 39 | / | / | / |
| 2 | Healthy volunteers | Male | 51 | / | / | / |
| 3 | Healthy volunteers | Female | 26 | / | / | / |
| 4 | Healthy volunteers | Male | 62 | / | / | / |
| 5 | Healthy volunteers | Female | 31 | / | / | / |
| 6 | Healthy volunteers | Male | 71 | / | / | / |
| 7 | Healthy volunteers | Male | 28 | / | / | / |
| 8 | Healthy volunteers | Female | 27 | / | / | / |
| 9 | Healthy volunteers | Male | 44 | / | / | / |
| 10 | Healthy volunteers | Male | 69 | / | / | / |
| 11 | Healthy volunteers | Female | 30 | / | / | / |
| 12 | Healthy volunteers | Male | 58 | / | / | / |
| 13 | BCa patient | Male | 69 | T2 | G2 | MIBC |
| 14 | BCa patient | Male | 58 | Ta | G1 | NMIBC |
| 15 | BCa patient | Female | 70 | T1 | G1 | NMIBC |
| 16 | BCa patient | Male | 66 | T1 | G3 | NMIBC |
| 17 | BCa patient | Female | 61 | T3 | G2 | MIBC |
| 18 | BCa patient | Male | 55 | T2 | G3 | MIBC |
| 19 | BCa patient | Male | 73 | T2 | G2 | MIBC |
| 20 | BCa patient | Female | 78 | T4 | G3 | MIBC |
| 21 | BCa patient | Female | 60 | T3 | G3 | MIBC |
| 22 | BCa patient | Male | 64 | T1 | G1 | NMIBC |
| 23 | BCa patient | Male | 52 | T2 | G2 | MIBC |
| 24 | BCa patient | Female | 71 | T4 | G3 | MIBC |
| 25 | BCa patient | Male | 59 | T1 | G1 | NMIBC |
| 26 | BCa patient | Male | 64 | T2 | G2 | MIBC |
| 27 | BCa patient | Male | 69 | T3 | G3 | MIBC |
| 28 | BCa patient | Female | 70 | T3 | G3 | MIBC |
| 29 | BCa patient | Male | 73 | T1 | G1 | NMIBC |
| 30 | BCa patient | Female | 65 | T2 | G2 | MIBC |
| 31 | BCa patient | Female | 62 | T3 | G3 | MIBC |
| 32 | BCa patient | Male | 74 | Ta | G1 | NMIBC |
| 33 | BCa patient | Male | 68 | T2 | G2 | MIBC |
| 34 | BCa patient | Female | 60 | T1 | G1 | NMIBC |
| 35 | BCa patient | Male | 80 | T4 | G3 | MIBC |
| 36 | BCa patient | Female | 77 | T2 | G2 | MIBC |
| 37 | BCa patient | Male | 62 | T1 | G1 | NMIBC |
| 38 | BCa patient | Male | 66 | T4 | G3 | MIBC |
| 39 | BCa patient | Male | 69 | T1 | G1 | NMIBC |
| 40 | BCa patient | Male | 64 | T2 | G2 | MIBC |
| 41 | BCa patient | Male | 62 | T1 | G1 | NMIBC |
| 42 | BCa patient | Female | 75 | T4 | G3 | MIBC |
| 43 | BCa patient | Male | 63 | Ta | G1 | NMIBC |
| 44 | BCa patient | Female | 61 | T2 | G2 | MIBC |
| 45 | BCa patient | Male | 50 | T1 | G1 | NMIBC |
| 46 | BCa patient | Male | 83 | T3 | G3 | MIBC |

**Supplementary Table S4. The clinicopathological information of Cohort 4.**

| **Patient number** | **Gender** | **Age** | **Tumour stage** | **Tumour Grade** | **Infiltration** |
| --- | --- | --- | --- | --- | --- |
| 1 | Male | 71 | T4 | G3 | MIBC |
| 2 | Female | 68 | T3 | G3 | MIBC |
| 3 | Male | 76 | T3 | G3 | MIBC |
| 4 | Male | 49 | T3 | G3 | MIBC |
| 5 | Female | 61 | T1 | G1 | NMIBC |
| 6 | Female | 66 | T2 | G1 | MIBC |
| 7 | Male | 64 | T3 | G3 | MIBC |
| 8 | Female | 53 | T1 | G3 | NMIBC |
| 9 | Male | 68 | T4 | G3 | MIBC |
| 10 | Male | 75 | T3 | G2 | MIBC |
| 11 | Female | 65 | T1 | G1 | NMIBC |
| 12 | Male | 59 | T2 | G1 | MIBC |
| 13 | Male | 66 | T3 | G3 | MIBC |
| 14 | Female | 57 | T2 | G1 | MIBC |
| 15 | Male | 68 | T2 | G3 | MIBC |
| 16 | Male | 71 | T4 | G2 | MIBC |
| 17 | Male | 59 | T4 | G2 | MIBC |
| 18 | Male | 77 | T1 | G2 | NMIBC |
| 19 | Male | 73 | T3 | G3 | MIBC |
| 20 | Male | 62 | T1 | G1 | NMIBC |

**Supplementary Table S5. The clinicopathological information of Cohort 5.**

| **No.** | **Category** | **Gender** | **Age** | **Tumour stage** | **Tumour Grade** | **Infiltration** |
| --- | --- | --- | --- | --- | --- | --- |
| 1 | Healthy volunteers | Male | 39 | / | / | / |
| 2 | Healthy volunteers | Male | 51 | / | / | / |
| 3 | Healthy volunteers | Female | 26 | / | / | / |
| 4 | Healthy volunteers | Male | 62 | / | / | / |
| 5 | Healthy volunteers | Female | 31 | / | / | / |
| 6 | Healthy volunteers | Male | 71 | / | / | / |
| 7 | Healthy volunteers | Male | 28 | / | / | / |
| 8 | Healthy volunteers | Female | 27 | / | / | / |
| 9 | Healthy volunteers | Male | 44 | / | / | / |
| 10 | Healthy volunteers | Male | 69 | / | / | / |
| 11 | Healthy volunteers | Female | 30 | / | / | / |
| 12 | Healthy volunteers | Male | 58 | / | / | / |
| 13 | Healthy volunteers | Male | 42 | / | / | / |
| 14 | Healthy volunteers | Male | 25 | / | / | / |
| 15 | Healthy volunteers | Female | 55 | / | / | / |
| 16 | Healthy volunteers | Female | 30 | / | / | / |
| 17 | Healthy volunteers | Male | 47 | / | / | / |
| 18 | Healthy volunteers | Male | 25 | / | / | / |
| 19 | Healthy volunteers | Female | 58 | / | / | / |
| 20 | Healthy volunteers | Male | 60 | / | / | / |
| 21 | BCa patient | Female | 68 | T2 | G2 | MIBC |
| 22 | BCa patient | Male | 64 | T1 | G1 | NMIBC |
| 23 | BCa patient | Male | 73 | T3 | G3 | MIBC |
| 24 | BCa patient | Female | 77 | T1 | G1 | NMIBC |
| 25 | BCa patient | Male | 58 | T2 | G3 | MIBC |
| 26 | BCa patient | Male | 69 | T2 | G2 | MIBC |
| 27 | BCa patient | Male | 58 | Ta | G1 | NMIBC |
| 28 | BCa patient | Female | 70 | T1 | G1 | NMIBC |
| 29 | BCa patient | Male | 66 | T1 | G3 | NMIBC |
| 30 | BCa patient | Female | 61 | T3 | G2 | MIBC |
| 31 | BCa patient | Male | 55 | T2 | G3 | MIBC |
| 32 | BCa patient | Male | 73 | T2 | G2 | MIBC |
| 33 | BCa patient | Female | 78 | T4 | G3 | MIBC |
| 34 | BCa patient | Female | 60 | T3 | G3 | MIBC |
| 35 | BCa patient | Male | 64 | T1 | G1 | NMIBC |
| 36 | BCa patient | Male | 52 | T2 | G2 | MIBC |
| 37 | BCa patient | Female | 71 | T4 | G3 | MIBC |
| 38 | BCa patient | Male | 59 | T1 | G1 | NMIBC |
| 39 | BCa patient | Male | 64 | T2 | G2 | MIBC |
| 40 | BCa patient | Male | 69 | T3 | G3 | MIBC |
| 41 | BCa patient | Female | 70 | T3 | G3 | MIBC |
| 42 | BCa patient | Male | 73 | T1 | G1 | NMIBC |
| 43 | BCa patient | Female | 65 | T2 | G2 | MIBC |
| 44 | BCa patient | Female | 62 | T3 | G3 | MIBC |
| 45 | BCa patient | Male | 74 | Ta | G1 | NMIBC |
| 46 | BCa patient | Male | 68 | T2 | G2 | MIBC |
| 47 | BCa patient | Female | 60 | T1 | G1 | NMIBC |
| 48 | BCa patient | Male | 80 | T4 | G3 | MIBC |
| 49 | BCa patient | Female | 77 | T2 | G2 | MIBC |
| 50 | BCa patient | Male | 62 | T1 | G1 | NMIBC |
| 51 | BCa patient | Male | 66 | T4 | G3 | MIBC |
| 52 | BCa patient | Male | 69 | T1 | G1 | NMIBC |
| 53 | BCa patient | Male | 64 | T2 | G2 | MIBC |
| 54 | BCa patient | Male | 62 | T1 | G1 | NMIBC |
| 55 | BCa patient | Female | 75 | T4 | G3 | MIBC |
| 56 | BCa patient | Male | 63 | Ta | G1 | NMIBC |
| 57 | BCa patient | Female | 61 | T2 | G2 | MIBC |
| 58 | BCa patient | Male | 50 | T1 | G1 | NMIBC |
| 59 | BCa patient | Male | 83 | T3 | G3 | MIBC |
| 60 | BCa patient | Male | 71 | T4 | G3 | MIBC |
| 61 | BCa patient | Female | 68 | T3 | G3 | MIBC |
| 62 | BCa patient | Male | 76 | T3 | G3 | MIBC |
| 63 | BCa patient | Male | 49 | T3 | G3 | MIBC |
| 64 | BCa patient | Female | 61 | T1 | G1 | NMIBC |
| 65 | BCa patient | Female | 66 | T2 | G1 | MIBC |
| 66 | BCa patient | Male | 64 | T3 | G3 | MIBC |
| 67 | BCa patient | Female | 53 | T1 | G3 | NMIBC |
| 68 | BCa patient | Male | 68 | T4 | G3 | MIBC |
| 69 | BCa patient | Male | 75 | T3 | G2 | MIBC |
| 70 | BCa patient | Female | 65 | T1 | G1 | NMIBC |
| 71 | BCa patient | Male | 59 | T2 | G1 | MIBC |
| 72 | BCa patient | Male | 66 | T3 | G3 | MIBC |
| 73 | BCa patient | Female | 57 | T2 | G1 | MIBC |
| 74 | BCa patient | Male | 68 | T2 | G3 | MIBC |
| 75 | BCa patient | Male | 71 | T4 | G2 | MIBC |
| 76 | BCa patient | Male | 59 | T4 | G2 | MIBC |
| 77 | BCa patient | Male | 77 | T1 | G2 | NMIBC |
| 78 | BCa patient | Male | 73 | T3 | G3 | MIBC |
| 79 | BCa patient | Male | 62 | T1 | G1 | NMIBC |
| 80 | BCa patient | Female | 67 | T1 | G1 | NMIBC |
| 81 | BCa patient | Female | 61 | T2 | G2 | MIBC |
| 82 | BCa patient | Male | 77 | T1 | G1 | NMIBC |
| 83 | BCa patient | Male | 58 | T2 | G1 | MIBC |
| 84 | BCa patient | Male | 70 | T3 | G3 | MIBC |
| 85 | BCa patient | Male | 65 | T3 | G3 | MIBC |
| 86 | BCa patient | Female | 68 | T4 | G3 | MIBC |
| 87 | BCa patient | Male | 74 | Ta | G1 | NMIBC |
| 88 | BCa patient | Male | 60 | T2 | G2 | MIBC |

**Supplementary Table S6. List of primers for qRT‒PCR.**

| **Gene name** | **Symbol** | **Forward primer** | **Reverse primer** | **Annealing Temperature (°C)** | **Length (bp)** |
| --- | --- | --- | --- | --- | --- |
| [Ankyrin repeat and SOCS box containing 12](https://www.ncbi.nlm.nih.gov/gene/142689) | *ASB12* | AGCAGTGTATGACAACGACTCC | AAGTGGCGTCTGTGCCTTG | 59 | 208 |
| TATA-box binding protein associated factor 12 | *TAF12* | TCTCATCCATAAAACCGGAACCA | TTCAGGGCTAAGACGACCTCC | 58 | 116 |
| [Karyopherin subunit alpha 3](https://www.ncbi.nlm.nih.gov/gene/3839) | *KPNA3* | ACAGTGGAACTGCGGAAGAAC | TCAATCGGTGGATTTCTGTCAC | 59 | 227 |
| Glutamate ionotropic receptor delta type subunit 2 | *GRID2* | TTTGTCCGTCTGGTGGTCTC | CAGTGCGAAATACCTCATCATCC | 57 | 113 |
| Lymphocyte antigen 6 family member G5B | *LY6G5B* | GATGTCAAGGTTCGCTTCATCG | AGGTGTTGCGTTTTTCTTGGC | 58 | 76 |
| Ectonucleoside triphosphate diphosphohydrolase 7 | *ENTPD7* | GCTTCATTACCACGAGATAGGC | TGATGTCCAGCAAGTCATGGG | 58 | 187 |
| [Stromal antigen 2](https://www.ncbi.nlm.nih.gov/gene/10735) | *STAG2* | TCCTTCTGGTCCAAACCGAAT | ACCGACTGCATAGCACTCTTG | 58 | 105 |
| [Vesicle associated membrane protein 2](https://www.ncbi.nlm.nih.gov/gene/6844) | *VAMP2* | CTCAAGCGCAAATACTGGTGG | TGATGGCGCAAATCACTCCC | 58 | 67 |
| MAM domain containing glycosylphosphatidylinositol anchor 2 | *MDGA2* | CTTTGACCATACCTGCCATCAC | ATTTTCACCTCACGGCCAATC | 57 | 190 |
| Valosin containing protein lysine methyltransferase | *VCPKMT* | GTGCGAGTTTTGGAGAAGCG | TCCTCAAGATCGGTGACTACAA | 57 | 248 |
| Cytochrome P450 family 3 subfamily A member 4 | *CYP3A4* | AAGTCGCCTCGAAGATACACA | AAGGAGAGAACACTGCTCGTG | 57 | 174 |
| Reticulon 4 interacting protein 1 | *RTN4IP1* | TGCCTGCTTGGGTGATAGATA | TGGCAGCGTGAACTTTGACA | 56 | 117 |
| Dysbindin domain containing 2 | *DBNDD2* | ATGGACCCAAATCCTCGGG | TGTCCTCGAAGAATTTTTGCCG | 56 | 76 |
| Rho related BTB domain containing 1 | *RHOBTB1* | ATGGACGCTGACATGGACTAC | ATCCCGAGAACGCTCCAAGA | 57 | 213 |
| GPN-loop GTPase 1 | *GPN1* | ACAGATTGAGGTATTCACCTGGT | GGTCACTGGGTTGGTACTTCT | 56 | 121 |
| Synaptotagmin 9 | *SYT9* | CTCTTTGGCGTGTCTCTCTTC | CTCCTGGTTGTTGTCTTTGCTA | 56 | 96 |
| Zinc finger protein 81 | *ZNF81* | GCAGTGCCTGTGAGGTATCAG | CGTCTTTGAGTAGAGTCCAGTTG | 57 | 91 |
| Fc alpha and mu receptor | *FCAMR* | GAGAAGTGGACTACTCCAGGC | GCGAAAGAAGAACCTTGTAGCAG | 57 | 124 |
| Progesterone receptor | *PGR* | ACCCGCCCTATCTCAACTACC | AGGACACCATAATGACAGCCT | 58 | 133 |
| Trophoblast glycoprotein | *TPBG* | ACCTCTTCCTTACCGGCAAC | CCCGAGAAAGCGAAGGGAC | 58 | 208 |
| Double PHD fingers 2 | *DPF2* | GGAGAATGTAGTGAAGCTCCTTG | CGAGCATTGTAATTGTGGCAC | 56 | 75 |
| Autophagy related 7 | *ATG7* | CAGTTTGCCCCTTTTAGTAGTGC | CCAGCCGATACTCGTTCAGC | 57 | 82 |
| Cyclin dependent kinase 12 | *CDK12* | CTAACAGCAGAGAGCGTCACC | AAAGGTTTGATAACTGTGCCCA | 57 | 121 |
| Tubulin beta 3 class III | *TUBB3* | GGCCAAGGGTCACTACACG | GCAGTCGCAGTTTTCACACTC | 58 | 85 |
| AT-rich interaction domain 2 | *ARID2* | CAGTGTGTCGGATTATCTGCG | GCATGACGTGCTTGCTTTCATT | 57 | 167 |
| Laminin subunit gamma 2 | *LAMC2* | GACAAACTGGTAATGGATTCCGC | TTCTCTGTGCCGGTAAAAGCC | 58 | 98 |
| Zinc finger MIZ-type containing 1 | *ZMIZ1* | TGTTTGACGGTGGTCAGTCG | CTTGTCTCGGTTTGCAGCAC | 58 | 96 |
| UV radiation resistance associated | *UVRAG* | GGCGTCTTCGACATCTTCGG | GACGGTCTGGCATAATTCCAAA | 57 | 198 |
| Actinin alpha 4 | *ACTN4* | GCAGCATGGGCGACTACAT | TTGAGCCCGTCTCGGAAGT | 58 | 172 |
| Progestin and adipoQ receptor family member 3 | *PAQR3* | AACCCGTACATCACCGACG | TCTGGACGCACTTGCTGAAG | 57 | 180 |
| Glyceraldehyde-3-phosphate dehydrogenase | *GAPDH* | GAAGGTGAAGGTCGGAGTC | GAAGATGGTGATGGGATTTC | 56 | 197 |

**Supplementary Table S7. List of primary antibodies.**

| **Antigens** | **Species antibodies raised in** | **Dilution (WB)** | **Dilution (IHC)** | **Supplier** |
| --- | --- | --- | --- | --- |
| HSP90, human | Rabbit, monoclonal | 1:10,000 | - | Abcam, UK, Cat. #ab203126 |
| HSP70, human | Mouse, monoclonal | 1:10,00 | - | Abcam, UK, Cat. #ab2787 |
| TSG101, human | Rabbit, monoclonal | 1:20,00 | - | Abcam, UK, Cat. #ab125011 |
| GAPDH, human | Mouse, monoclonal | 1:2,000 | - | Santa Cruz Biotechnology Inc., USA, Cat. #sc-365062 |
| ASB12, human | Rabbit, Polyclonal | - | 1:100 | Novus Biologicals, USA, Cat. #NBP2-46775 |
| E-Cadherin, human | Rabbit, monoclonal | - | 1:5,000 | Abcam, UK, Cat. #ab76055 |
| Ki-67, human | Rabbit, monoclonal | - | 1:200 | Novus Biologicals, USA, Cat. #NBP2-19012 |

**Supplementary Table S8. List of secondary antibodies and counterstaining of nuclei.**

| **Secondary detection system used** | **Host** | **Method** | **Dilution** | **Supplier** |
| --- | --- | --- | --- | --- |
| Anti-Mouse-IgG (H+L)-HRP | Goat | WB | 1:10,000 | Sungene Biotech, China, Cat. #LK2003 |
| Anti-Rabbit-IgG (H+L)-HRP | Goat | WB | 1:10,000 | Sungene Biotech, China, Cat. #LK2001 |
| Anti-rabbit IgG (H+L), F(ab')2 Fragment (Alexa Fluor® 488 Conjugate) | Goat | WB | 1:50 | Cell Signaling Technology, USA, Cat. #4412 |
| Anti-mouse IgG (H+L), F(ab')2 Fragment (Alexa Fluor® 555 Conjugate) | Goat | WB | 1:50 | Cell Signaling Technology, USA, Cat. #4408 |
| Anti-goat IgG-FITC | Rabbit | IF | 1:100 | Boster Biological Technology, China, Cat. #BA1110 |
| Anti-goat IgG-Cy3 | Rabbit | IF | 1:100 | Boster Biological Technology, China, Cat. #BA1034 |
| Hoechst 33342 nucleic acid staining (DAPI) | - | IF | 1:750 | Molecular Probes/Invitrogen, USA, Cat. #A11007 |

**Supplementary Table S9. List of 35 upregulation lncRNAs.**

| **Target ID** | **Probe Name** | **fold Change** | **Ave Expr** | **t** | **P.Value** | **B** |
| --- | --- | --- | --- | --- | --- | --- |
| ENST00000453784 | A_33_P3559138 | 23.77 | 9.8957 | 2.3685 | 0.0313 | -4.5400 |
| lnc-TAF12-2:1 | A_22_P00015744 | 11.12 | 8.5057 | 2.1658 | 0.0464 | -4.5491 |
| ENST00000500036 | CUST_14740_PI437845250111 | 8.88 | 7.4529 | 2.1929 | 0.0440 | -4.5479 |
| ENST00000548057 | CUST_14469_PI437845250111 | 8.5 | 10.9557 | 2.2505 | 0.0394 | -4.5453 |
| ENST00000548475 | CUST_14446_PI437845250111 | 8.37 | 10.8071 | 2.3087 | 0.0352 | -4.5427 |
| ENST00000458044 | A_21_P0001246 | 8.09 | 7.0886 | 2.1483 | 0.0479 | -4.5499 |
| ENST00000561316 | A_22_P00016181 | 6.97 | 9.5443 | 2.3762 | 0.0308 | -4.5397 |
| lnc-PYROXD2-1:1 | CUST_4792_PI437944915 | 6.86 | 8.1871 | 2.1445 | 0.0483 | -4.5500 |
| ENST00000592121 | CUST_18156_PI437845250111 | 5.94 | 8.3586 | 2.1357 | 0.0491 | -4.5504 |
| ENST00000603261 | CUST_8390_PI437845250111 | 5.06 | 7.3486 | 2.1950 | 0.0438 | -4.5478 |
| ENST00000623792 | A_33_P3235282 | 5.02 | 7.0771 | 2.3426 | 0.0329 | -4.5412 |
| NONHSAT214233.1 | CUST_19658_PI437845424 | 5.01 | 8.4286 | 2.2198 | 0.0418 | -4.5467 |
| NONHSAT190443.1 | CUST_11202_PI437845424 | 4.95 | 7.3957 | 2.1353 | 0.0491 | -4.5504 |
| ENST00000513026 | CUST_7412_PI437845250111 | 4.91 | 9.0043 | 2.3196 | 0.0344 | -4.5422 |
| NONHSAT192849.1 | CUST_11812_PI437845424 | 4.84 | 7.6443 | 2.2494 | 0.0395 | -4.5454 |
| lnc-ITIH1-1:1 | CUST_720_PI437845420 | 4.71 | 8.4629 | 2.3581 | 0.0319 | -4.5405 |
| NONHSAT214359.1 | CUST_19723_PI437845424 | 4.7 | 6.9643 | 2.3277 | 0.0339 | -4.5419 |
| lnc-C12orf42-3:1 | A_21_P0007750 | 4.53 | 6.2500 | 2.1950 | 0.0438 | -4.5478 |
| NONHSAT173753.1 | CUST_5580_PI437845424 | 4.46 | 7.2043 | 2.3971 | 0.0296 | -4.5388 |
| lnc-FOXA2-3:1 | A_21_P0010112 | 4.42 | 6.9571 | 2.2991 | 0.0358 | -4.5431 |
| ENST00000445310 | CUST_8424_PI437845250 | 4.41 | 8.0957 | 2.2762 | 0.0375 | -4.5442 |
| lnc-LILRB1-2:4 | CUST_7708_PI437859739 | 4.34 | 7.6543 | 2.2721 | 0.0378 | -4.5443 |
| NONHSAT172402.1 | CUST_5212_PI437845424 | 4.32 | 5.9657 | 2.4054 | 0.0291 | -4.5384 |
| ENST00000430772 | CUST_20750_PI437845250111 | 4.31 | 8.7514 | 2.2076 | 0.0428 | -4.5472 |
| lnc-GPR128-2:1 | CUST_908_PI437845420 | 4.2 | 7.8771 | 2.3051 | 0.0354 | -4.5429 |
| lnc-SPAG5-2:2 | CUST_5192_PI437859739 | 4.17 | 8.5557 | 2.3596 | 0.0318 | -4.5404 |
| lnc-MYL12B-3:1 | CUST_6165_PI437859739 | 4.16 | 7.1143 | 2.2245 | 0.0414 | -4.5465 |
| lnc-DUSP28-2:1 | CUST_10152_PI437859739 | 3.41 | 7.2729 | 2.2403 | 0.0402 | -4.5458 |
| lnc-CCL2-8:1 | CUST_5287_PI437859739 | 3.39 | 7.0300 | 2.2510 | 0.0393 | -4.5453 |
| lnc-TOMM22-1:1 | CUST_11656_PI437859739 | 3.05 | 6.7057 | 2.2973 | 0.0360 | -4.5432 |
| lnc-RP11-80A15.1.1-5 | A_21_P0008503 | 2.96 | 6.0945 | 2.1845 | 0.0302 | -4.7521 |
| lnc-BTNL8-5 | CUST_17771_PI437845420 | 2.77 | 7.5691 | 2.3520 | 0.0321 | -4.8109 |
| lnc-N6AMT2-3 | CUST_16506_PI437845420 | 2.59 | 8.2764 | 2.2880 | 0.0361 | -4.9270 |
| lnc-ZNF256-2 | CUST_7789_PI437859739 | 2.38 | 7.3827 | 2.3650 | 0.0377 | -4.9688 |
| lnc-AFP-1 | CUST_2299_PI437845420 | 2.14 | 6.0936 | 2.5302 | 0.0402 | -4.0320 |

**Supplementary Table S10. List of top predicted miRNA for lnc-TAF12-2:1 by applying three independent databases.**

| **Target Rank** | **miRNA** | **Target Score** | **Correlation** | **P.Value** |
| --- | --- | --- | --- | --- |
| 1 | miR-876-3p | 97 | -0.7867 | 0.0053 |
| 2 | miR-3149 | 95 | -0.7839 | 0.0066 |
| 3 | miR-4534 | 94 | -0.7684 | 0.0072 |
| 4 | miR-1827 | 94 | -0.7537 | 0.0107 |
| 5 | miR-7847-3p | 92 | -0.7460 | 0.0180 |
| 6 | miR-548b-3p | 91 | -0.7297 | 0.0251 |
